# *SYISL* Knockout Promotes Embryonic Muscle Development of Offspring by Modulating Maternal Gut Microbiota and Fetal Myogenic Cell Dynamics

**DOI:** 10.1002/advs.202410953

**Published:** 2024-12-16

**Authors:** Hao Zuo, Wei Jiang, Jianwei Gao, Zhibo Ma, Chen Li, Yaxin Peng, Jianjun Jin, Xizhen Zhan, Wei Lv, Xiao Liu, Jingjing Hu, Mengdi Zhang, Yiming Jia, Zaiyan Xu, Junming Tang, Rong Zheng, Bo Zuo

**Affiliations:** ^1^ Key Laboratory of Swine Genetics and Breeding of the Ministry of Agriculture and Rural Affairs Huazhong Agricultural University Wuhan 430070 China; ^2^ Key Laboratory of Agriculture Animal Genetics Breeding and Reproduction of the Ministry of Education Huazhong Agricultural University Wuhan 430070 China; ^3^ Hubei Hongshan Laboratory Wuhan 430070 China; ^4^ Department of Basic Veterinary Medicine College of Veterinary Medicine Huazhong Agricultural University Wuhan 430070 China; ^5^ Hubei Key Laboratory of Embryonic Stem Cell Research School of Basic Medicine Science Hubei University of Medicine Shiyan 442000 China

**Keywords:** butyrate, gut microbiota, muscle fiber number, scRNA‐seq, *SYISL*

## Abstract

Embryonic muscle fiber formation determines post‐birth muscle fiber totals. The previous research shows *SYISL* knockout significantly increases muscle fiber numbers and mass in mice, but the mechanism remains unclear. This study confirms that the *SYISL* gene, maternal gut microbiota, and their interaction significantly affect the number of muscle fibers in mouse embryos through distinct mechanisms, as *SYISL* knockout alters maternal gut microbiota composition and boosts butyrate levels in embryonic serum. Both fecal microbiota transplantation and butyrate feeding significantly increase muscle fiber numbers in offspring, with butyrate inhibiting histone deacetylases and increasing histone acetylation in embryonic muscle. Combined analysis of RNA‐seq between wild‐type and *SYISL* knockout mice with ChIP‐seq for H3K9ac and H3K27ac reveals that *SYISL* and maternal microbiota interaction regulates myogenesis via the butyrate‐HDAC‐H3K9ac/H3K27ac pathway. Furthermore, scRNA‐seq analysis shows that *SYISL* knockout alone significantly increases the number and proportion of myogenic cells and their dynamics, independently of regulating histone acetylation levels. Cell communication analysis suggests that this may be due to the downregulation of signaling pathways such as MSTN and TGFβ. Overall, multiple pathways are highlighted through which *SYISL* influences embryonic muscle development, offering valuable insights for treating muscle diseases and improving livestock production.

## Graphical abstract

*SYISL* knockout significantly promotes embryonic muscle development and increases muscle fiber numbers of offspring by modulating maternal gut microbiota composition and butyrate‐HDAC‐H3K9ac/H3K27ac pathway. Furthermore, scRNA‐seq analysis shows that *SYISL* knockout alone significantly increases the number and proportion of myogenic cells and their dynamics, which may be due to the downregulation of signaling pathways such as MSTN and TGFβ.

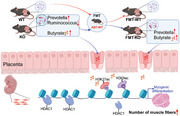

## 1 Introduction

Understanding the formation of muscle fibers is integral for developing methods of treating various muscle diseases for the improvement of meat production. The growth and development of skeletal muscle involve two stages: the formation of embryonic muscle fibers and the enlargement of muscle fibers after birth, in which the number of muscle fibers during the embryonic period determines the quantity of muscle fibers after birth.^[^ ^1^ ^]^ In the process of mouse embryonic development, primary muscle fibers, which predominantly express the slow myosin heavy chain (MyHC I) protein, are formed between embryonic days 11.5 and 14.5 (E11.5d–E14.5d). Subsequently, secondary muscle fibers are formed until E18.5d, and thereafter the number of muscle fibers remains stable.^[^ ^2^ ^]^ The development of embryonic muscle fibers is influenced by multiple factors such as transcription factors,^[^ ^3^ ^]^ epigenetic modifiers,^[^ ^4^ ^]^ and non‐coding RNAs.^[^ ^5^ ^]^ The absence of the transcription factor *KLF4* results in a significant reduction in both the weight of embryos at E17.5d and the area of muscle fibers.^[^ ^6^ ^]^ Knockout (KO) of *KDM4A*, a histone demethylase, inhibits myogenic differentiation by reducing the demethylation of the histone marker H3K9me3 at the regulatory regions of key myogenic genes such as *MyoD* and *MyoG*, leading to reduced embryonic weight and decreased muscle fiber size in mice.^[^ ^7^ ^]^

Recent studies have established key interaction relationship between host genes and gut microbes,^[^ ^8^ ^]^ revealing host genes can influence the composition of gut microbiota or their metabolic products,^[^ ^9^ ^]^ subsequently affecting muscle development.^[^ ^10^ ^]^ For example, *MSTN* deficiency leads to the enrichment of microbes in the colon that produce short‐chain fatty acids (SCFAs), thus increasing valeric acid production, which activates the Akt/mTOR pathway via G protein‐coupled receptor 43 (GPR43), eventually stimulating the growth of type IIb muscle fibers.^[^ ^11^ ^]^ Transplanting the microbiota of Rongchang pigs into germ‐free (GF) mice has been shown to alter mice muscle composition, resulting in an increase of the type I muscle fiber proportion and a reduction in the measured area of type IIb muscle fibers.^[^ ^12^ ^]^ SCFAs are the most extensively studied microbial metabolites in the gut‐muscle axis, with numerous SCFAs receptors such as GPR41 and GPR43 present in muscle tissue.^[^ ^13^ ^]^ SCFAs can affect skeletal muscle development by activating multiple signaling pathways, such as AMP‐activated protein kinase (AMPK), peroxisome proliferator‐activated receptor δ (PPAR‐δ), and peroxisome proliferator‐activated receptor‐γ coactivator‐1α (PGC‐1α), or by inhibiting histone deacetylases (HDACs) activity.^[^ ^14^ ^]^ SCFAs are small molecular substances, that can be transferred from the mother through the placenta to the fetus,^[^ ^15^ ^]^ while sources have also shown that adding sodium butyrate to pregnant rats can significantly reduce blood pressure and levels of inflammatory factors, and increase the weight of the fetus and placenta.^[^ ^16^ ^]^

Our previous studies identified Synaptopodin‐2 intron sense‐overlapping lncRNA (*SYISL*) as a novel suppressor of muscle growth in mice, pigs, and humans,^[^ ^17^ ^]^ and demonstrated that knockout of *SYISL* could result in a significant increase in the total number of muscle fibers and muscle mass in mice.^[^ ^18^ ^]^ However, how *SYISL* influences offspring embryonic myogenesis is largely unknown. In this study, we discovered that the interaction between maternal *SYISL* and gut microbes significantly increases the number of embryonic muscle fibers. Specifically, *SYISL* knockout significantly altered the composition of the maternal intestinal microbiota, leading to a significant increase in the abundance of *Prevotella* and butyrate‐producing bacteria, which in turn significantly raised the levels of butyrate in the maternal serum, feces, and fetal serum. Concurrently, we also discovered that butyrate was involved in the significant increase in the number of embryonic muscle fibers caused by the *SYISL* knockout through the HDAC‐H3K27ac/H3K9ac pathway. Moreover, single‐cell RNA sequencing (scRNA‐seq) of WT and KO fetal muscles from the same heterozygous parents showed that *SYISL* knockout leads to a significant increase in myogenic cells and the number of embryonic muscle fibers, likely through various key signaling pathways such as TGFβ, MSTN, and PTN. Our study reveals the multiple pathways by which *SYISL* regulates embryonic myogenesis in offspring.

## 2 Results

### 2.1 Interaction Between Maternal *SYISL* and Gut Microbes Significantly Affects Numbers of Secondary and Total Muscle Fibers in Offspring

**Figure 1:**
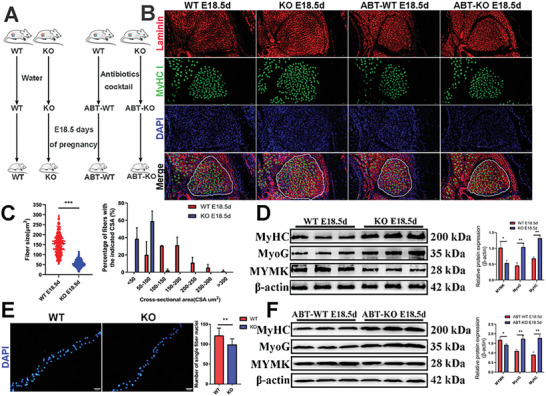
The interaction between host genes and gut microbes in female mice has significant effects on the number of muscle fibers in their offspring. A) After antibiotic treatment, female mice of either SPF or pseudo‐germ‐free were mated with male mice with the corresponding microbial status. Samples were collected on gestational day 18.5. *n* = 9. B) Representative images of immunofluorescence staining for laminin (red) and MyHC I (green) in the cross‐sections of the hind limbs of KO and WT fetuses at E18.5d. The white dashed lines delineate the mouse EDL muscle. Scale bars, 50 µm. *n* = 5. C) Analysis of EDL muscle fiber cross‐sectional areas in E18.5d mice. The left figure presents a scatter plot of the areas of each muscle fiber, and the right figure shows the distribution of the muscle fibers with different area ranges. Compared to WT mice, KO mice exhibit a higher proportion of muscle fibers with smaller cross‐sectional areas. In an independent experiment, the areas of 100 myofibers from the EDL muscle of each mouse were analyzed. Data were presented as mean ± SDs, *n* = 3. ***p < 0.001. D,F) Western blotting results showed that in both SPF (D) and ABT (F) mice, *SYISL* KO significantly increased the protein expression levels of MyoG and MyHC genes and notably decreased the protein expression levels of MYMK genes. Data were presented as mean ± SDs, *n* = 3. *p < 0.05, **p < 0.01. E) Representative images of DAPI staining of single myofibers and quantification of four independent experiments show that *SYISL* knockout significantly decreases the number of nuclei per fiber. Scale bar, 50 µm. Twenty single myofibers/muscle/mouse were analyzed in an independent experiment. Data were presented as mean ± SDs, **p < 0.01.

To determine interaction between host *SYISL* gene and gut microbiota and its potential effects on muscle fiber development in offspring, we prepared germ‐free WT and KO mice (ABT‐WT and ABT‐KO) and established four mating groups (**Figures** **1**A, S1A, Supporting Information). We performed immunofluorescence staining for laminin and MyHC I in the leg muscles of E18.5d embryos, and the number of muscle fibers in the extensor digitorum longus (EDL) muscles were counted (Figure 1B). The results showed a significant increase in the number of primary, secondary, and total muscle fibers in KO fetuses compared to WT fetuses (*p* < 0.05), along with a significant decrease in the muscle fiber area in KO fetuses (**Table** **1** and Figure 1C). KO fetuses showed higher protein expression levels of the differentiation marker MyoG and MyHC, but lower protein expression levels of the fusion marker MYMK in leg muscles than WT fetuses (Figure 1D). These results were consistent with those at postnatal day 0.5 (Figure S1B–E, Supporting Information). Additionally, DAPI staining of isolated individual muscle fibers showed that the number of nuclei per fiber in KO mice was significantly lower than that in WT mice (*p* < 0.01, Figure 1E).

**Table 1:** Analysis of muscle fiber number of four different types of fetuses at E18.5d.

Type	Primary muscle fibers number	Secondary muscle fiber number	Total muscle fiber number
WT E18.5d	164.75 ± 25.53^b^	605.75 ± 15.57^b^	770.5 ± 25.39^c^
KO E18.5d	204.8 ± 11.01^c^	799.4 ± 25.41^c^	1004.2 ± 33.88^d^
ABT‐WT E18.5d	103.25 ± 7.75^a^	446.5 ± 39.05^a^	549.75 ± 43.62^a^
ABT‐KO E18.5d	124.5 ± 8.79^a^	581.75 ± 22.61^b^	706.25 ± 28.10^b^

Values with the same number of letters indicate that the difference is not significant. Different letters indicate that the difference is significant. The data represent the means ± SDs, *n* = 5.

Immunofluorescence results showed that ABT‐WT fetuses exhibited a significant reduction in the number of primary, secondary, and total muscle fibers at E18.5d in comparison to untreated WT fetuses, and similar results were observed between ABT‐KO and untreated KO fetuses (*p* < 0.01, Table 1 and Figure 1B). Western blotting results revealed a significant decrease in the differentiation and fusion capabilities of embryonic skeletal muscle after antibiotics treatment (Figure S1F,G, Supporting Information). ABT‐KO fetuses also showed an increase in muscle fiber numbers compared to ABT‐WT mice (Table 1). The differences in primary, secondary, and total muscle fiber numbers between WT and KO fetuses were more prominent than between ABT‐KO and ABT‐WT fetuses (Table 1 and Figure 1B). The differences in MYMK, MyoG, and MyHC protein levels in leg muscles between WT and KO fetuses were also larger than those between ABT‐WT and ABT‐KO fetuses (Figure 1D,F). These results suggest that maternal gut microbiota had shown effects on embryonic muscle fiber formation. Two‐way analysis of variance (ANOVA) analysis of the above data further confirmed that the interaction between the maternal *SYISL* genotype and gut microbiota significantly affected on the numbers of secondary and total muscle fibers (*p* < 0.05), whereas the maternal *SYISL* genotype alone and gut microbiota alone affected the numbers of primary, secondary, and total muscle fibers (**Table** **2**). In summary, the *SYISL* gene, maternal gut microbiota, and their interaction had notable effects on the formation of embryonic muscle development.

**Table 2:** Results of two‐way ANOVA.

Type	Source of variance	Sum of squares	df	Mean square	F	*p*
Primary muscle fibers	Genotype	4440.2	1	4440.2	18.602	0.001**
	Gut microbe	25063.2	1	25063.2	104.999	<0.001***
	Genotype × Gut microbe	480.2	3	160.1	2.012	0.175
Secondary muscle fibers	Genotype	135136.8	1	135136.8	185.902	<0.001***
	Gut microbe	177472.8	1	177472.8	244.142	<0.001***
	Genotype × Gut microbe	4032.8	3	1344.3	5.548	0.032*
Total muscle fibers	Genotype	188568.2	1	188568.2	166.495	<0.001***
	Gut microbe	335923.2	1	335923.2	296.601	<0.001***
	Genotype × Gut microbe	7296.2	3	2432.1	6.442	0.022*

Two‐factor ANOVA was used to study the impact of genotype and gut microbiota on primary, secondary, and total muscle fibers. The data represent the means ± SDs of five independent experiments; *p < 0.05, **p < 0.01, ***p < 0.001.

### 2.2 *SYISL* Knockout Changes the Gut Microbiota Composition and Metabolites of Pregnant Mice

**Figure 2:**
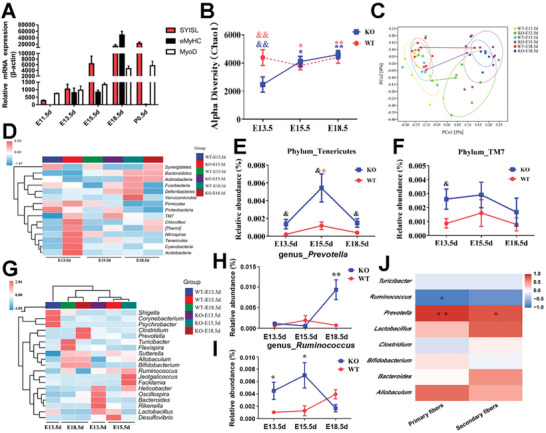
*SYISL* gene knockout in pregnant mice significantly affects the gut microbiota composition. A) RT‐qPCR results indicate that the expression of the *SYISL* gene increased with embryonic muscle development. *eMyHC* and *MyoD* were used as positive references. B) The Chao1 index indicates variations in the gut microbiota abundance of WT and KO female mice during different stages of pregnancy. During pregnancy, gut microbiota in WT mice exhibited a stable trend, while the abundance of gut microbiota in KO mice showed a significant increasing trend. The two groups of maternal mice only displayed significant differences in gut microbiota at E13.5d. C) PCoA results demonstrated significant differences in the gut microbiota of WT and KO maternal mice at gestational days 13.5, 15.5, and 18.5. *n* = 6. D,G) The heatmap of species clustering of fecal microbiota at the phylum (D) and genus (G) levels during different stages of pregnancy in WT and KO female mice is represented. E,F) The relative abundance changes of fecal microbiota in WT and KO female mice during all three stages of pregnancy for microbial groups phylum *Tenericutes* (E), TM7 (F). At three stages, the abundance of phylum *Tenericutes* and *TM7* in KO mice was higher than that in the WT group. H,I) Relative abundance changes in the taxonomic composition of *Prevotella* (H) and *Ruminococcus* (I) at the genus level in WT and KO maternal mice at gestational days 13.5, 15.5, and 18.5. J) The correlation heatmap showing the correlation results among abundance of *Prevotella* and *Ruminococcus*, and number of primary and secondary muscle fibers at E18.5d. The data represent the means ± SDs of three independent experiments; *p < 0.05, **p < 0.01.

Since RT‐qPCR results showed that *SYISL* gene expression significantly increased from E13.5 to E18.5d (**Figure** **2**A), we collected female mouse fecal samples at E13.5d, E15.5d, and E18.5d from WT and KO mice and analyzed the composition of the fecal microbiota by 16S rRNA sequencing. These results showed that at E13.5d, the Chao1 index was significantly lower in the KO group in the WT group (*p* < 0.01). As pregnancy progressed, the Chao1 index in the KO group significantly increased at E15.5d compared to E13.5d (*p* < 0.05), and both the WT and KO groups exhibited significantly higher Chao1 index (*p* < 0.01) and Shannon index at E18.5d than at E13.5d (*p* < 0.05, Figure 2B; Figure S2A, Supporting Information). Principal coordinate analysis (PCoA) showed that during all three stages of pregnancy (E13.5, E15.5, and E18.5d), the fecal microbiota of the KO group significantly separated from that of the WT group (*p* < 0.05, Figure 2C). As pregnancy progressed, the microbial structure in the feces of the WT mice remained relatively stable (*p* > 0.05, Figure S2B, Supporting Information), whereas that of KO mice exhibited significant differences across the three pregnancy stages (*p* < 0.05, Figure S2C, Supporting Information). These results suggesting that *SYISL* KO might substantially alter the composition of the microbiota with a significant change occurring at E15.5d (secondary fiber formation stage). Our analysis of the bacterial differential abundance at the phylum showed that during all three stages of pregnancy, the abundance of *Tenericutes* and *TM7* was significantly higher in the KO female mice than in WT (*p* < 0.05, Figure 2D–F, Figure S2D, Supporting Information), whereas the abundance of *Proteobacteria* was significantly lower in the KO female mice than in WT (Figure S2E, Supporting Information). Furthermore, we performed genus‐level analysis (Figure 2G, Figure S2F, Supporting Information) and found that the relative abundance of *Prevotella* significantly increased (*p* < 0.01, Figure 2H), whereas that of the genus *Ruminococcus* significantly decreased in the KO group (*p* < 0.05, Figure 2I). We validated the accuracy of the 16S rRNA results by RT‐qPCR analysis of fecal DNA samples from WT and KO mice collected at E18.5d (Figure S3A,B, Supporting Information), and our data showed significant differences in the relative abundance of the *Prevotella* and *Ruminococcus* genera during the critical period of secondary muscle fiber development (E15.5d–E18.5d). These findings suggest that two genera mentioned might play important roles in muscle fiber development.

We conducted Spearman correlation analyses to assess the relationship between the relative abundance of intestinal genera (with >1% abundance) and the number of embryonic muscle fibers at E18.5d. The results showed a significant positive correlation between *Prevotella* and the number of both primary and secondary muscle fibers (*p* < 0.05), and a significant negative correlation between *Ruminococcus* and the number of primary muscle fibers (*p* < 0.05, Figure 2J). In summary, *SYISL* knockout led to significant changes in the composition of the host gut microbiota and promoted an increase in the abundance of *Prevotella*.

**Figure 3:**
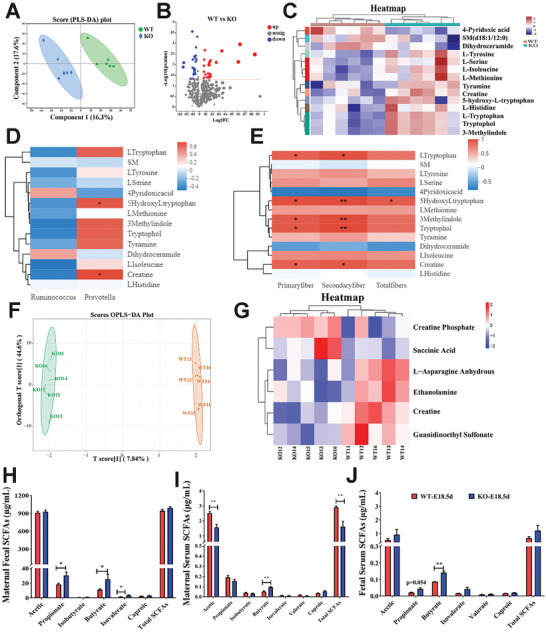
*SYISL* knockout in female mice significantly alters the abundance of endogenous metabolites. A) PLS‐DA analysis revealed significant differences in metabolite profiles between KO and WT mice. *n* = 6. B) Volcano plots of differential metabolites in fecal samples from WT and KO female mice show a total of 62 differential metabolites, with 34 metabolites significantly upregulated and 28 significantly downregulated in the KO group compared to the WT group. C) A heatmap illustrates the differential metabolites in fecal samples from WT and KO mice. D) Correlation analysis between differential metabolites in fecal samples and core differential genera (*Prevotella, Ruminococcus*) was performed. E) Correlation analysis demonstrated relationships between differential metabolites in fecal samples and embryonic muscle fiber number in the mice. F) The differences in serum metabolite profiles dependent on the genotype of maternal mice were analyzed using Orthogonal Partial Least Squares Discriminant Analysis (OPLS‐DA), revealing significant variations between the metabolites of KO and WT groups. *n* = 6. G) A heatmap shows differentially abundant serum metabolites in KO and WT maternal mice, with two metabolites significantly upregulated and four metabolites significantly downregulated in the KO group compared to the WT group. H,I) Quantification of SCFAs in maternal mouse feces (H) and serum (I) at gestational day 18.5 showed significant increases in propionate, butyrate, and isovalerate levels in the feces of KO maternal mice. In serum, there was a significant increase in butyrate levels, while acetic acid and total SCFAs exhibited significant decreases. Data were presented as mean ± SDs, *n* = 3. *p < 0.05. **p < 0.01. J) Quantification of SCFAs levels in the serum of E18.5 fetuses showed a significant increase only in butyrate. Data were presented as mean ± SDs, *n* = 3. *p < 0.05. **p < 0.01.

To reveal the metabolic changes caused by alterations in the gut microbiota, we analyzed the fecal samples of WT and KO mice at gestational day 18.5 using liquid chromatography‒mass spectrometry (LC‒MS). Using partial least squares discriminant analysis (PLS‐DA), we observed significant differences in the metabolite profiles between WT and KO (**Figure** **3**A). In the comparison between KO and WT specimens, a total of 62 metabolites with differential abundance were identified, among which 34 were upregulated and 28 were downregulated (Figure 3B). Kyoto Encyclopedia of Genes and Genomes (KEGG) pathway enrichment analysis indicated that these differential metabolites were mainly enriched in the pathways associated with protein degradation, protein transport, and amino acid metabolism (Figure S3C, Supporting Information). Differential metabolite heatmap showed that compared to those in the WT group, some metabolites such as Creatine, L‐Histidine, L‐Serine, L‐Isoleucine, L‐Tryptophan, L‐Methionine, Tryptophol, and 3‐Methylindole significantly increased in the KO group, while metabolites such as 4‐Pyridoxic acid, SM (d18:1/12:0), and Dihydroceramide were significantly decreased (Figure 3C). Next, we conducted correlation analysis between these fecal metabolites and gut microbiota associated with muscle fiber development. There were significant positive correlations between *Prevotella* and metabolites such as 5‐Hydroxy‐L‐tryptophan (5‐HTP) and creatine (*p* < 0.05, Figure 3D). Following these results, Spearman correlation analysis between differentially abundant metabolites and muscle fiber number was performed (Figure 3E). Therefore, the results show that such metabolites significantly positively correlated with embryonic muscle fiber number included tryptophan, 5‐HTP, 3‐methylindole, tryptophol, creatine (*p* < 0.05, Figure 3E). Serum profiling analysis revealed significant differences between WT and KO female mice at 18.5d of pregnancy (Figure 3F). Compared to the WT group, the KO group showed a significant increase in the levels of creatine phosphate and succinic acid (*p* < 0.05), but a significant decrease in the levels of L‐asparagine anhydrous, ethanolamine, guanidinoethyl sulfonate, and creatine (*p* < 0.05, Figure 3G).

SCFAs are crucial microbiota metabolic products, which can be transported to the fetus via the placenta.^[^ ^13^ ^]^ In this study, we examined SCFAs in the feces and serum of female mice at 18.5d of pregnancy and fetal serum at E18.5d. Compared to WT female mice, KO female mice showed significant increases in the levels of propionate, butyrate, and isovalerate in fecal samples (Figure 3H), and *SYISL* KO female mice exhibited an elevated level of butyric acid but reduced levels of acetic acid and total SCFAs in serum samples (Figure 3I). Spearman correlation analysis reveals a very strong correlation between maternal fecal butyrate and secondary muscle fibers in the fetus, as well as a notable correlation with the total muscle fiber count. Propionate also shows a notable correlation with secondary muscle fibers (Figure S3D, Supporting Information). In maternal serum, butyrate is positively correlated with both secondary and total muscle fiber counts, while caproic acid has a notable correlation with secondary fibers (Figure S3E, Supporting Information). In *SYISL* KO fetal serum, only the level of butyrate was significantly elevated (Figure 3J). We also observed a significant increase in the abundance of butyrate‐producing bacteria such as *Clostridium cluster XIVa* and *Faecalibacterium prausnitzii* (Figure S3F, Supporting Information). In conclusion, we came to the finding that *SYISL* knockout leads to an increase in the abundance of SCFAs‐producing bacteria such as *Prevotella*, *Clostridium cluster XIVa*, and *Faecalibacterium prausnitzii*, thus increasing the content of butyric acid in maternal and fetal serum.

### 2.3 Transplantation of KO Female Mouse Fecal Microbiota to ABT Mice Results in Increased Offspring Muscle Fibers

**Figure 4:**
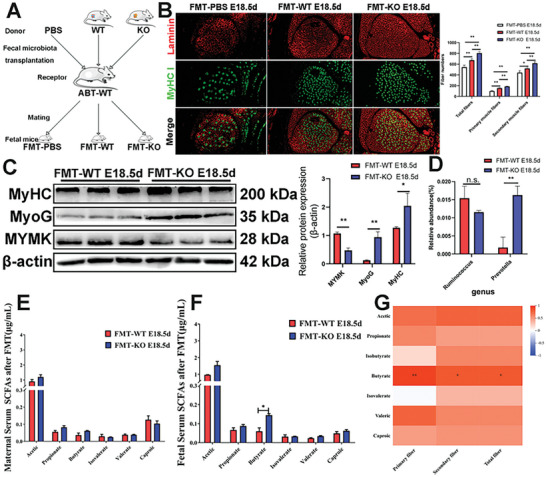
FMT of KO fecal microbiota in ABT mice results in more muscle fibers in offspring than that of WT fecal microbiota. A) ABT‐WT mice were subjected to 14 d of gavage with PBS, WT, and KO fecal microbiota suspensions. Subsequently, all female mice were mated with WT male mice, and samples were collected from female mice at gestational day 18.5. *n* = 9. B) Representative images of immunofluorescence staining for laminin (red) and MyHC I (green) in the fetus EDL at E18.5d from FMT‐PBS, FMT‐WT, and FMT‐KO groups. Quantification analysis showed FMT‐KO group had the highest number of primary, secondary, and total muscle fibers. White dashed lines delineate the EDL muscle. Scale bar, 50 µm. *n* = 6. C) Western blotting results showed that MyoG and MyHC protein expression levels of fetus muscles at 18.5d from FMT‐KO group were significantly higher than those from FMT‐WT group, but MYMK protein expression level of fetus muscles at 18.5d from FMT‐KO group was significantly lower than those from FMT‐WT group. Data were presented as mean ± SDs, *n* = 3. *p < 0.05, **p < 0.01. D) Column chart results indicate that compared to the FMT‐WT group, there was no significant change in the relative abundance of gut *Ruminococcus* of FMT‐KO mice at gestational day 18.5, but there was a significant increase in the relative abundance of gut *Prevotella*. Data were presented as mean ± SDs, *n* = 3. n.s. not significant, **p < 0.01. E,F) Quantification of SCFAs revealed that there was an upward trend of propionic, acetic, and butyrate in the serum of FMT‐KO mice at gestational day 18.5 (E) and a significant elevation of butyrate in the fetus serum at E18.5d from FMT‐KO group (F), compared to FMT‐WT groups. G) Heatmap representation showing the correlation results of SCFAs in FMT‐KO embryonic serum with the number of embryonic muscle fibers. The data represent the means ± SDs of three independent experiments.

Fecal microbiota transplantation (FMT) experiments involve transferring donor gut microbiota from WT and KO female mice, as well as phosphate‐buffered saline (PBS), into the gastrointestinal tract of ABT‐WT recipient mice (**Figure** **4**A). At E18.5d, fetal leg muscles were collected for immunofluorescence staining. The results showed that compared to FMT‐PBS fetuses, both FMT‐KO and FMT‐WT fetuses exhibited a significant increase in the muscle fiber quantity. Specifically, compared to FMT‐WT fetuses, FMT‐KO fetuses showed increase in primary, secondary, and total muscle fiber numbers by 19.31%, 16.78%, and 17.36%, respectively (Figure 4B). Western blotting results showed that FMT‐KO fetuses exhibited significantly higher protein expression levels of the differentiation markers MyHC and MyoG in the leg muscles compared to FMT‐WT fetuses, but a significantly lower of the fusion protein MYMK (Figure 4C). Overall, the FMT experiments indicated that transplanting the gut microbiota from KO mice into ABT‐KO mother mice significantly increased the number of muscle fibers and related phenotypes in their embryos.

Fecal samples were collected from FMT‐WT and FMT‐KO female mice at 18.5d of pregnancy and subjected to 16S rRNA sequencing; the results showed significant differences in the microbial composition between the two groups. Through microbiota profiling, a total of 4757 operational taxonomic units (OTUs) were identified, of which 716 OTUs were shared by both groups; 756 OTUs were unique to FMT‐WT female mice; and 3285 OTUs were unique to FMT‐KO female mice (Figure S4A, Supporting Information), although no significant differences in the Chao1 and Shannon indices between FMT‐WT and FMT‐KO female mice (*p* > 0.05, Figure S4B, Supporting Information) were found. PCoA results showed that the fecal microbiota of the FMT‐KO group was significantly separated from that of the FMT‐WT group (Figure S4C, Supporting Information). At the phylum level, the predominant microbial phyla in the FMT‐KO fecal microbiota included *Bacteroidetes*, *Firmicutes, Proteobacteria*, and *Actinobacteria*, of which *Firmicutes* and *Bacteroidetes* accounted for more than 85% of the total abundance (Figure S4D, Supporting Information). At the genus level (Figure S4E, Supporting Information), significant differences in microbial composition were observed between the FMT‐WT and FMT‐KO groups. The relative abundance of *Prevotella* was significantly increased in FMT‐KO mouse feces (*p* < 0.01), whereas that of *Ruminococcus* was decreased in FMT‐KO mouse feces, but not statistically significant (*p* > 0.05, Figure 4D). Further, the accuracy of the 16S rRNA results was validated by RT‐qPCR analysis of extracted fecal DNA from FMT mice at day 18.5 of pregnancy (Figure S4F,G, Supporting Information). These results might be attributed to the reshaping of the intestinal microbiota spatial structure during FMT. The SCFAs was measured in maternal and fetal serum, where an increase in the levels of both acetic acid and butyrate was observed in maternal serum (Figure 4E), while a significant increase in the level of butyrate only was observed in fetal serum (Figure 4F). The correlation analysis results indicate that butyrate in the embryonic serum is highly correlated with the number of primary muscle fibers in the fetus (*p* < 0.01). Additionally, it shows strong correlations with both the number of secondary and total muscle fibers (*p* < 0.05, Figure 4G), further validating that butyrate might play an important role in embryonic muscle fiber development.

### 2.4 Butyrate Significantly Increases Offspring Muscle Fiber Number and Inhibits Offspring Muscle HDACs Activity

**Figure 5:**
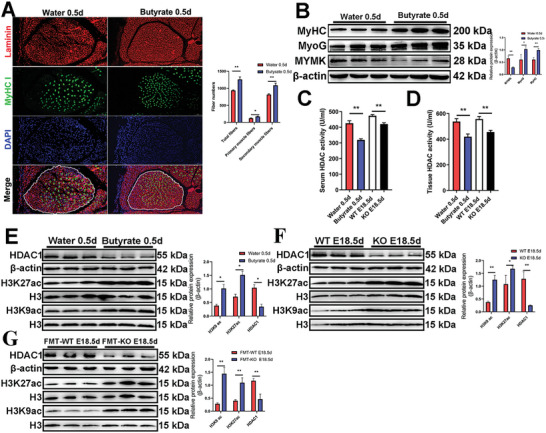
Feeding butyrate to female mice significantly increased the offspring muscle fiber number and inhibited muscle HDACs activity. A) Representative images of immunofluorescence staining for laminin (red) and MyHC I (green) in the EDL from 0.5d (post‐birth) old water‐fed and butyrate‐fed mice. The quantitative analysis revealed that the number of primary, secondary, and total muscle fibers was significantly higher in mice drinking water supplemented with 0.02% sodium butyrate compared to control mice drinking plain water. White dashed lines delineate the EDL muscle. Scale bar, 50 µm. Data were presented as mean ± SDs, *n* = 6. *p < 0.05, **p < 0.01. B) Western blotting results showed that 0.02% sodium butyrate significantly increased the protein levels of MyoG and MyHC, along with a notable decrease MYMK protein in the leg muscle. Data were presented as mean ± SDs, *n* = 3. *p < 0.05, **p < 0.01. C,D) HDACs activity in the serum (C) and leg muscle (D) from the offspring of butyrate‐fed mice at 0.5d and KO fetuses at E18.5d were significantly lower compared to their respective control groups. Data were presented as mean ± SDs, *n* = 3. **p < 0.01. E–G) Western blotting analysis showed that the offspring of 0.02% butyrate‐fed mice (E), *SYISL* KO fetuses (F), and FMT‐KO fetuses (G) had lower HDAC1 protein expression and higher H3K27ac and H3K9ac protein levels in leg muscle compared to their respective control groups. Protein levels were normalized to control β‐actin levels. Data were presented as mean ± SDs, *n* = 3. *p < 0.05, **p < 0.01.

To investigate the potential effects of butyrate on embryonic muscle development, we fed female mice with diets containing sodium butyrate. Immunofluorescence staining of the leg muscles collected at postnatal day 0.5 revealed a significant increase in the numbers of primary (*p* < 0.05), secondary (*p* < 0.01), and total (*p* < 0.01) muscle fibers in sodium butyrate group compared to control group (**Figure** **5**A). Western blotting experiments revealed that sodium butyrate feeding promoted the expression of MyHC and MyoG proteins but suppressed the expression of MYMK protein in the offspring's leg muscles (Figure 5B). Since butyrate is a well‐known inhibitor of HDACs, we examined HDACs activity in 0.5‐day‐old butyrate‐fed and control mice, as well as KO and WT fetuses at E18.5d. As expected, a significant decrease in HDACs activity was observed in the serum and leg muscle of butyrate‐fed 0.5‐day‐old mice and KO fetuses, compared to the control group (Figure 5C,D). Next, we examined the expression of HDAC1 protein and histone acetylation levels in fetal muscles of butyrate‐fed and control mice. The results showed that the expression of HDAC1 protein was notably lower in the butyrate‐fed group versus the control group, while the expressions of H3K9ac and H3K27ac were significantly higher (Figure 5E). Similar results were observed in fetal leg muscles of KO (Figure 5F) and FMT‐KO (Figure 5G) mice at E18.5d. Overall, the results indicated that butyrate significantly inhibited HDACs activity and increased the levels of H3K9ac and H3K27ac, thereby increasing the number of muscle fibers in the offspring.

### 2.5 Butyrate Regulates the Host *SYISL* Knockout‐Mediated Myogenesis Through HDAC‐H3K9ac/H3K27ac Pathway

**Figure 6:**
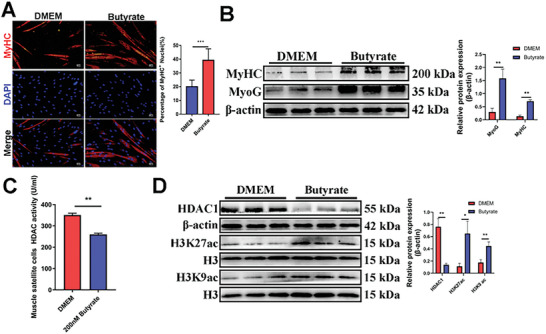
200 nm butyrate significantly promotes myogenic differentiation, suppresses the activity of HDACs and increases H3K9ac and H3K27ac levels in skeletal muscle satellite cells. A) Representative images of immunofluorescence staining for MyHC in differentiated mouse skeletal muscle satellite cells and quantification showed that butyrate significantly promoted myoblast differentiation. Scale bars, 100 µm. Data were presented as mean ± SDs, *n* = 3. ***p < 0.001. B) Western blotting results showed that treatment of mouse skeletal muscle satellite cells with 200 nm butyrate significantly increased the protein levels of MyoG and MyHC. Data were presented as mean ± SDs, *n* = 3. **p < 0.01. C) Mouse skeletal muscle satellite cells were treated with 200 nm butyrate and then induced into differentiation, and the supernatant was subjected to an HDACs activity assay. The quantification showed treatment with butyrate significantly increased HDACs activity. Data were presented as mean ± SDs, *n* = 3. **p < 0.01. D) Western blotting results showed that treatment of mouse skeletal muscle satellite cells with 200 nm butyrate inhibits HDAC1 protein expression and increases the protein levels of H3K27ac and H3K9ac. The relative protein levels were normalized to those of the control β‐actin and H3. Data were presented as mean ± SDs, *n* = 3. *p < 0.05, **p < 0.01.

To further investigate the possible mechanism by butyrate in the promotion of myogenesis, we treated C2C12 cells with different concentrations of sodium butyrate and examined their differentiation status. RT‐qPCR and Western blotting results showed that 200 nm sodium butyrate substantially promoted the expression of MyoG and MyHC (Figure S5A–C, Supporting Information). MyHC immunofluorescence staining results revealed that the addition of 200 nm sodium butyrate notably enhanced myogenic differentiation in C2C12 cells (Figure S5D, Supporting Information). Similar results were also observed in immunofluorescence and Western blotting assays of mouse skeletal muscle satellite cells treated with the same concentration of sodium butyrate (**Figure** 6A,B). We also observed that 200 nm sodium butyrate treatment significantly decreased HDACs activity in muscle satellite cells and C2C12 cells (Figure 6C; Figure S5E, Supporting Information), and this treatment significantly increased the levels of H3K9ac and H3K27ac while significantly decreased HDAC1 protein levels (Figure 6D; Figure S5F, Supporting Information). In summary, butyrate inhibited HDACs activity to increase acetylation levels and enhanced the differentiation of both muscle satellite cells and C2C12 cells.

**Figure 7:**
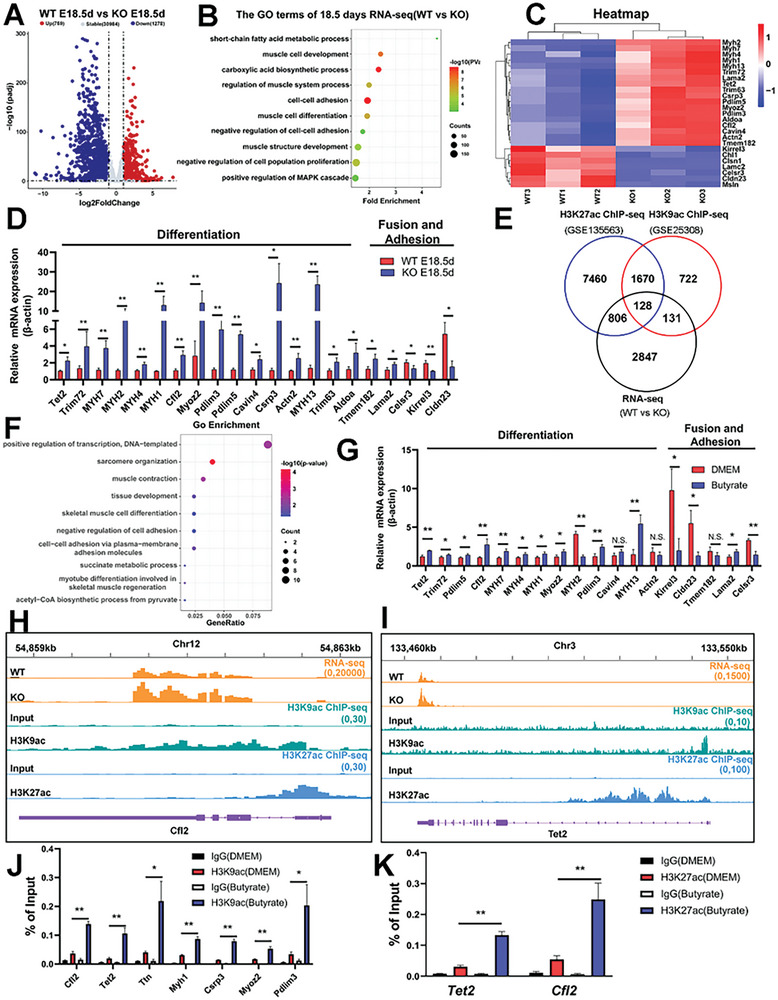
Butyrate participates in the host *SYISL* knockout‐mediated myogenesis through HDAC‐H3K9ac/H3K27ac pathway. A) Volcano plot of RNA‐seq data showing DEGs in leg muscle from WT mice and KO mice at E18.5d. The DEGs were determined by adjusting *p‐*value <0.05 and absolute FC > 1.5. A total of 2037 DEGs were identified, of which 1278 genes were downregulated and 759 genes were upregulated. *n* = 3. B) GO enrichment analysis revealed that the 2037 differentially expressed genes were primarily enriched in processes related to muscle tissue development, cell adhesion, muscle cell differentiation, and SCFAs metabolism. C) Heatmap of differentially expressed muscle cell differentiation and adhesion‐related genes. D) RT‐qPCR was performed to validate the DEGs involved in muscle cell differentiation, fusion, and adhesion when β‐actin was used as the reference gene, and the results were consistent with the sequencing data. Data were presented as mean ± SDs, *n* = 3. *p < 0.05, **p < 0.01. E) Overlapping analysis among RNA‐seq (WT vs KO), H3K9ac ChIP‐seq (GEO number: GSE25308) and H3K27ac ChIP‐seq (GEO number: GSE135563) in ESCs. F) Enrichment analysis of 128 genes between RNA‐seq, H3K9ac ChIP‐seq, and H3K27ac ChIP‐seq indicated these genes were mainly related to cell differentiation and cell adhesion. G) RT‐qPCR was performed to validate the RNA‐seq DEGs related to muscle cell differentiation and adhesion in mouse muscle satellite cells treated with 200 nm butyrate for 3 days, when β‐actin was used as the reference gene. Data were presented as mean ± SDs, *n* = 3. n.s. not significant, *p < 0.05, **p < 0.01. H,I) Combined profiles of RNA‐seq (orange), H3K9ac (green), H3K27ac (blue) ChIP‐seq profiles for *Cfl2* (H), *Tet2* (I). J,K) ChIP‐qPCR results showed the H3K9ac (J) and H3K27ac (K) enrichments at the promoter regions of myogenic regulatory factors were significantly increased in mouse muscle satellite cells treated with 200 nm butyrate for 3 days. IgG was used as negative control. Data were presented as mean ± SDs, *n* = 3. *p < 0.05, **p < 0.01.

Further, we first performed RNA sequencing of muscles from WT and KO fetuses at E18.5d. Principal component analysis (PCA) results revealed that significant differences in gene expression between WT and KO fetuses (Figure S6A, Supporting Information). In the comparison between WT and KO, a total of 2037 differentially expressed genes (DEGs) were identified, with 759 upregulated and 1278 downregulated in KO mouse muscle tissues (**Figure** **7**A). Gene ontology (GO) enrichment analysis showed that these DEGs were mainly enriched in biological processes related to muscle tissue development, cell adhesion, muscle cell differentiation, and SCFAs metabolism (Figure 7B). Gene set enrichment analysis (GSEA) revealed that *SYISL* knockout significantly affected the pathways related to HDAC deacetylate histones, cell‐cell junction organization, and regulation of actin cytoskeleton (Figure S6B–D, Supporting Information). In KO mouse muscles, the expressions of fusion and adhesion‐related genes like *Chl1, Celsr3, kirrel3, Cldn23* significantly decreased, while those of genes associated with the muscle cell differentiation such as *Tet2, MYH7, MYH1, Cfl2*, and *Actn2* significantly increased (Figure 7C). RT‐qPCR was conducted to validate the RNA‐seq results, with expression changes in DEGs including *MYH7*, *MYH4*, *MYH1*, *Cfl2*, and *Tet2*. RT‐qPCR results were consistent with RNA‐seq data, confirming the accuracy of the RNA‐seq results (Figure 7D). Furthermore, we intersected our dataset of identified DEGs in the leg muscles of 18.5‐day embryos and the two public ChIP‐seq datasets of H3K9ac (GSE25308) and H3K27ac (GSE135563) in embryonal stem cells (Figure 7E). A total of 128 common genes were identified across the three datasets and GO enrichment analysis showed that these shared genes were mainly involved in cell differentiation and cell adhesion (Figure 7F). Afterward, GO enrichment analysis also showed that the 259 overlapping genes between the RNA‐seq and H3K9ac ChIP‐seq datasets were mainly related to cell adhesion, cell migration, muscle differentiation, and metabolic pathways (Figure S6E, Supporting Information), and 934 overlapping genes between the RNA‐seq and H3K27ac ChIP‐seq datasets were mainly associated with cell adhesion, stem cell differentiation, and muscle organ development (Figure S6F, Supporting Information). Subsequently, RT‐qPCR and ChIP‐qPCR were performed on mouse skeletal muscle satellite cells after 200 nm sodium butyrate treatment. As expected, RT‐qPCR results showed that sodium butyrate treatment significantly increased the expression of genes related to muscle differentiation such as *Cfl2*, *Tet2*, *Myh7*, and *Myh4*, and decreased the expression of genes promoting cell fusion such as *Kirrel3*, *Cldn23*, and *Celsr3* (Figure 7G). ChIP‐qPCR results showed that H3K9ac and H3K27ac were significantly enriched in the regulatory regions of myogenic genes such as *Cfl2* and *Tet2* in mouse muscle satellite cells after 200 nm butyrate treatment (Figure 7H–K, Figure S7, Supporting Information), indicating that butyrate treatment upregulated myogenic gene expression by increasing the enrichment of H3K9ac and H3K27ac in the regulatory regions of these genes, promoting muscle generation. In summary, butyrate regulates muscle generation mediated by *SYISL* knockout through the HDAC‐H3K9ac/H3K27ac pathway.

### 2.6 The scRNA‐Seq Analysis Revealed that the deletion of the *SYISL* Gene Significantly Increases the Number of Fetal Myogenic Cells and Promotes Their Differentiation

**Figure 8:**
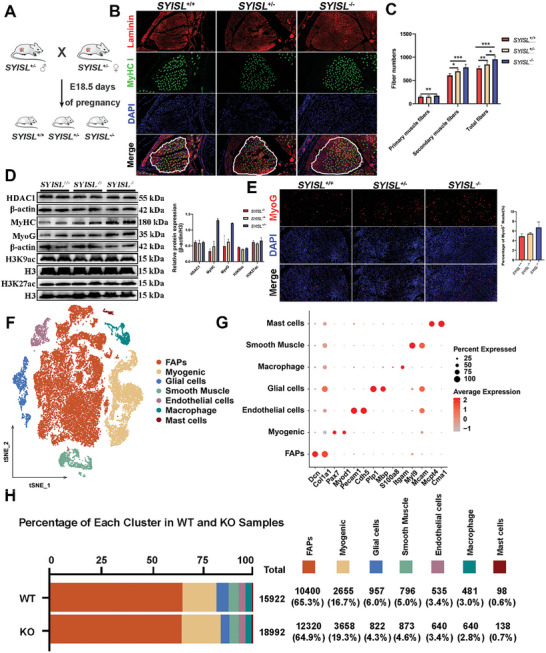
Changes in muscle fiber number and various cell types in the muscles of fetal mice with three different genotypes from the same litter. A) Schematic representation of E18.5d fetal mouse samples with different genotypes obtained from mating *SYISL* heterozygous mice. B) Representative images of immunofluorescence staining for laminin (red) and MyHC I (green) in the cross‐sections of the hind limbs of *SYISL* ^+/+^, *SYISL* ^−/+^ and *SYISL* ^−/‐^ fetuses at E18.5d. The white dashed lines delineate the mouse EDL muscle. Scale bars, 50 µm. *n* = 3. C) Quantitative analysis of immunofluorescence results. D) Western blotting results showed that compared with WT (*SYISL* ^+/+^) fetuses, the protein expression levels of MyoG and MyHC genes in muscles were upregulated in *SYISL* ^±^ and *SYISL* ^−/‐^ fetuses at E18.5d, while there were no changes at HDAC1 protein and histone acetylation (H3K9ac, H3K27ac) levels among them. Data were presented as mean ± SDs, *n* = 2. E) Representative images of immunofluorescence staining for MyoG in both *SYISL* ^+/+^, *SYISL* ^−/+^and *SYISL* ^−/‐^ fetuses at E18.5d and quantification analysis of immunofluorescence results showed that compared with *SYISL* ^+/+^ fetuses, MyoG protein expression in muscles was significantly upregulated *SYISL* ^±^ and *SYISL* ^−/‐^ fetuses. Scale bars, 100 µm. Data were presented as mean ± SDs, *n* = 2 F) t‐SNE visualization of the single‐cell transcriptional profiling of leg muscles derived from WT and KO mice, with colors delineating different cell types, seven cell populations: FAPs; Myogenic; Endothelial cells; Glial cells; Macrophages; Smooth Muscle; Mast cells (*n* = 2/each group). G) Bubble chart illustrating the expression of marker genes used for cell type annotation. Marker genes include: FAPs (*Dcn, Col1a1*); Myogenic (*Pax7, Myod1*); Endothelial cells (*Pecam1, Cdh5*); Glial cells (*Plp1, Mbp*); Macrophages (*Itgam*); Smooth Muscle (*My19, Mcam*); Mast cells (*Mcpt4, Cma1*). The color of each circle reflects the average expression level of the marker genes, while the size indicates the percentage of cells expressing those genes. H) Bar graph displaying the proportion of different cell types across various samples, with distinct colors representing different cell types.

To further verify the importance role of *SYISL* alone on embryonic myogenesis, we mated *SYISL* heterozygous mice and collected fetal samples at E18.5d (**Figure** **8**A), thereby excluding maternal effect. Immunofluorescence staining of the leg muscles revealed that the numbers of primary, secondary, and total muscle fibers in KO mice were significantly higher than those in their WT littermates. Similarly, the numbers of secondary and total muscle fibers in *SYISL* heterozygous mice were also significantly higher compared to WT mice (Figure 8B,C). Western blotting results showed that alterations in the *SYISL* gene did not affect HDAC1 or histone acetylation levels, while the differentiation marker proteins MyHC and MyoG were still significantly elevated after *SYISL* knockout samples (Figure 8D). Immunofluorescence experiments further confirmed that *SYISL* knockout promoted MyoG expression (Figure 8E). These results suggest that *SYISL* influences embryonic muscle development independently of the HDAC‐H3K9ac/H3K27ac pathway.

To further explore the mechanism of *SYISL* knockout on muscle fiber development, we performed 10X Genomics scRNA‐seq on leg muscles from E18.5d WT and KO fetal mice from the same litter. After excluding low‐quality cells, a total of 34 914 cells were included in the analysis, including 15 922 cells from WT samples and 18 992 from KO samples. Through unsupervised clustering analysis based on marker gene expression, we identified seven cell populations: FAPs, Myogenic, Endothelial cells, Glial cells, Macrophages, Smooth Muscle cells, and Mast cells (Figure 8F,G; Figure S8A, Supporting Information). The relatively uniform distribution of these populations across samples suggests minimal batch effects within specific clusters (Figure S8B, Supporting Information). Notably, the numbers of FAPs and myogenic cells were significantly increased in the KO samples compared to the WT samples, particularly the myogenic cells, which showed the most substantial change when supported with statistical analysis (Figure 8H). Immunofluorescence results further confirmed a significant increase in myogenic cells in KO samples, potentially linked to enhanced muscle fiber formation (Figure 8E; Figure S8C, Supporting Information). In summary, significant increases of muscle fiber numbers and the number/proportion of myogenic cells were found in WT and KO fetal mice from the same litter.

### 2.7 Pseudotemporal and Cell Communication Analysis Indicates that *SYISL* KO May Alter the Dynamics of Myogenic Cells by Modifying Signaling Pathways Such as MSTN

**Figure 9:**
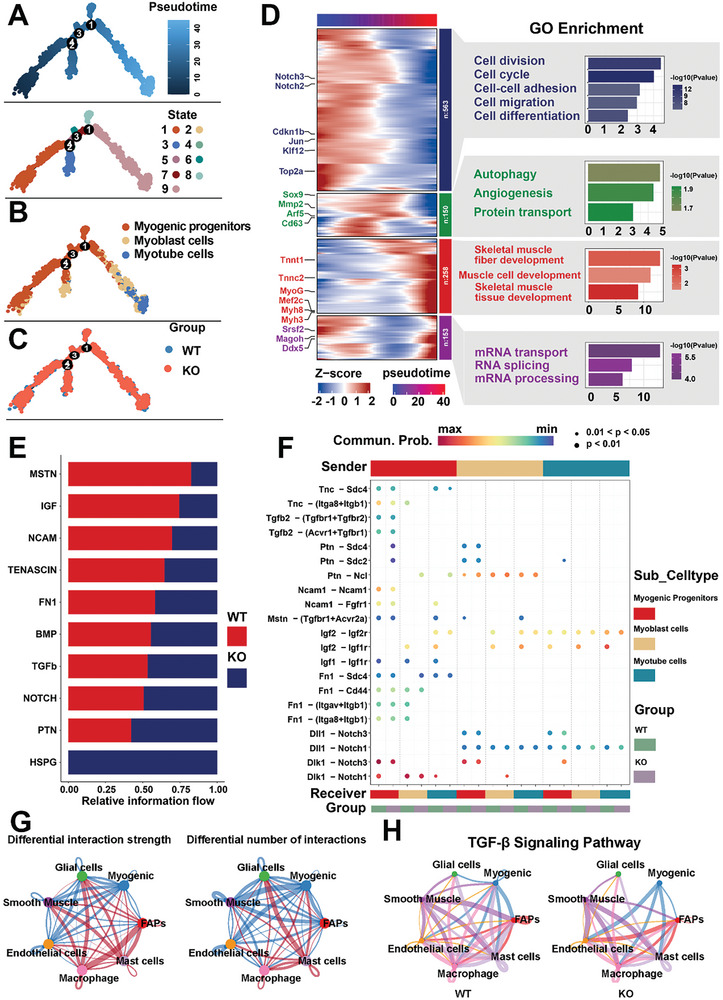
Analysis of pseudotemporal and CellChat revealed that *SYISL* knockout promotes the number and differentiation of myogenic cells by possibly affecting the TGFβ and MSTN signaling pathways. A) Pseudotime analysis of myogenic cells was performed by Monocle 2 and revealed nine different cell states. The distributions of cell states were presented along with pseudotime flows. Each dot is a cell. Black dots indicate significant differentiation branch points. B) Pseudotime developmental trajectory shows the developmental trajectory of myogenic cells from the progenitor stage to the mature myotube stage. Red dots represent myogenic progenitor cells, orange dots indicate myoblasts, and blue dots depict myotubes. C) The pseudotime analysis plot compares the developmental trajectories of myogenic cells in the WT and KO groups, with blue representing WT cells and red representing KO cells. D) Heatmap illustrating the dynamic expression of differentially expressed genes during the pseudotime trajectory of cell differentiation from myogenic progenitors to myotubes, conducted specifically within myogenic cells. Gene expression data are organized into four clusters based on k‐means clustering. The right panel lists GO terms enriched for each gene set, highlighting key biological processes involved in each cluster. E) Bar graph illustrating differential signaling pathways between WT and KO samples within myogenic cells. Each bar represents the relative information flow through various pathways, with red bars indicating WT and blue bars representing KO. F) Dotplot shows the communication probabilities of ligand‐receptor pairs across different cellular subtypes, as derived from CellChat analysis. Dot colors range from purple (minimum) to red (maximum) to represent the strength of communication, while dot sizes indicate statistical significance. Cellular subtypes are distinguished by color: myogenic progenitors (red), myoblasts (yellow), and myotubes (blue). Additionally, genetic groups (WT and KO) are shown to assess the impact of genotype on intercellular communication. G) This network diagram is used to compare the changes in interaction strength and quantity among different cell types between WT and KO samples. The left panel presents the changes in interaction strength, and the right panel illustrates the variation in the number of interaction strengths. Blue lines indicate decreased interaction strength in KO compared to WT, while red lines show increased interaction strength. Nodes are color‐coded to represent different cell types. H) Communication networks of TGFβ pathways analyzed using CellChat. Each node represents a distinct cell type, and the connecting lines indicate potential interactions.

To further investigate how *SYISL* knockout influences myogenic cell composition, we utilized the Monocle 2 algorithm to analyze their pseudotime trajectories, categorizing the cells into nine stages from early (dark blue) to later stages (light blue) (**Figure** **9**A). We then grouped these nine states into three main myogenic subgroups based on gene expression profiles: myogenic progenitors, myoblasts, and myotubes (Figure S8D,E Supporting Information). The developmental trajectory charts reveal multiple critical junctures in the transition from myogenic progenitors to myotubes, highlighting the complex and dynamic regulation of muscle development (Figure 9B). To explore gene expression dynamics along the developmental trajectories between WT and KO myogenic cells, we labeled KO cells in red and WT cells in blue (Figure 9C). The pseudotime analysis heatmap indicated that, compared to WT samples, KO cells exhibited higher expression of genes like *Notch2* and *Notch3* in early myogenesis stages, which decreasing in later stages. In contrast, differentiation marker genes such as *MyoG*, *Myh8*, and *Myh3* are significantly upregulated in later stages (Figure 9D). GO enrichment analysis indicated that these differential genes are primarily involved in the regulation of skeletal muscle development, cell proliferation, differentiation, and protein transport (Figure 9D).

To uncover the underlying cell communication changes caused by *SYISL* knockout, we used CellChat to assess potential intercellular interactions among myogenic cells, where we discovered that *SYISL* knockout leads to significant changes in key signaling pathways involved in muscle development. Specifically, signaling pathways such as *MSTN* and its receptors (*Tgfbr1+Acvr2a*), *Igf2*, *Igf1*, and their common receptor *Igf1r*, were markedly reduced post‐knockout. Conversely, the interaction between *PTN* and its receptors (*Sdc4* and *Sdc2*) was significantly enhanced. Additionally, pathways like TGFβ and HSPG were altered (Figure 9E,F). Muscle tissue is a highly heterogeneous tissue, which results in myogenic cells closely interacting with other cells during development. Analyzing myogenic cells alongside other cell types revealed significant changes in communication patterns within the KO group (Figure 9G). After *SYISL* knockout, the reception of classical signaling pathways, such as TGFβ, by myogenic cells from other cell groups was altered (Figure 9H; Figure S8F,G, Supporting Information). These findings suggest that alterations in signaling pathways like MSTN and TGFβ may play a crucial role in *SYISL*’s regulation of the number and state of myogenic cells.

## 3 Discussion

Increasing evidence indicates that there is a complex interaction between host genes and gut microbiota, playing a significant role in organism development.^[^ ^8^, ^19^ ^]^ In this study, we found that the *SYISL* gene, maternal gut microbiota, and their interaction all significantly affect the number of embryonic muscle fibers. *SYISL* knockout significantly changed the composition of the maternal mouse gut microbiota, and increased the abundance of *Prevotella* as well as butyrate‐producing bacteria. Correlation analysis revealed significant association of the abundance of *Prevotella* and *Ruminococcus* genera with the number of embryonic muscle fibers. Similar associations have been noted in *MSTN* KO cattle, which have higher skeletal muscle mass and greater intestinal *Prevotella* abundance than WT cattle.^[^ ^20^ ^]^ Conversely, a significant decrease in *Prevotella* abundance has been reported in patients with muscle‐wasting disorders.^[^ ^21^ ^]^ Additionally, we observed an increase in the abundance of *Prevotella* in the feces of FMT‐KO maternal mice, whose embryo also had more muscle fibers. Therefore, the increase in muscle fiber numbers in fetuses might be partially attributed to the high abundance of *Prevotella* in the maternal gut microbiota.

*Prevotella* is a core genus in various organisms, including humans and pigs, capable of degrading complex food components to produce a variety of degrading enzymes such as cellulases, mannanases, and xylanases.^[^ ^22^ ^]^ In pigs with a *Prevotella*‐dominated enterotype, a significant increase in the abundance of the butyrate‐producing bacteria, *Clostridium cluster XIVa*, enhances the butyrate content within the organism.^[^ ^23^ ^]^ Adding butyrate to a high‐fat diet can increase the percentage of type I muscle fibers in mouse skeletal muscle.^[^ ^24^ ^]^ Butyrate also helps prevent muscle atrophy and loss associated with aging or diseases through various mechanisms.^[^ ^25^ ^]^ Although butyrate has a significant impact on muscle mass and type transformation, there are no reports regarding its effect on the number of muscle fibers. Therefore, this study confirms that KO‐pregnant mice exhibited higher butyrate levels in feces and serum compared to WT controls. Importantly, this increase was positively correlated with an elevated number of muscle fibers in their fetuses, as supplementing maternal mice with sodium butyrate significantly increased the number of muscle fibers in the offspring, providing further evidence to support this correlation. Additionally, butyrate can specifically inhibit HDACs activity in tissue‐ or cell‐type‐dependent manner.^[^ ^26^ ^]^ β‐Hydroxybutyrate, a butyrate analog, inhibits HDAC1 activity in muscle stem cells, promoting p53 acetylation, leading to deep quiescence and enhanced elasticity of these cells.^[^ ^27^ ^]^ We found that the HDACs activity in the fetal muscle of KO mice, FMT‐KO mice, and butyrate‐fed mice significantly decreased, and the levels of histone acetylation significantly increased. Consistently, we observed significant enrichment of H3K9ac and H3K27ac in the regulatory regions of myogenic genes such as *Cfl2* and *Tet2* in muscle satellite cells after butyrate treatment, leading to upregulated expression of these genes. Notably, among these genes, *Tet2*, a key enzyme in DNA demethylation of 5‐methylcytosine, is crucial for muscle development. *Tet2* knockout in mice can significantly decrease the muscle fiber number.^[^ ^28^ ^]^ The observed increase in muscle fibers after *SYISL* knockout suggests a possible link to elevated *Tet2* expression; however, further studies are needed to confirm this relationship.

Our metabolomic data indicated that metabolite levels such as creatine and 5‐Hydroxy‐L‐tryptophan positively correlate with embryonic muscle fiber numbers, with creatine levels being notably higher in the feces of KO pregnant mice compared to WT. Creatine has been reported to enhance the differentiation of myogenic C2C12 cells by activating both *p38* and Akt/PKB pathways.^[^ ^29^ ^]^ In this study, *SYISL* knockout resulted in a significant increase in the serum succinate levels in pregnant mice. Succinate, a metabolite produced by *Prevotella*, is a crucial substance in the tricarboxylic acid cycle.^[^ ^30^ ^]^ Our results show that succinate metabolism and related pathways were enriched in the DEGs when comparing WT to KO mice, as succinate plays a crucial role in the conversion of skeletal muscles from fast‐twitch to slow‐twitch^[^ ^31^ ^]^; whether this metabolite affects muscle fiber numbers remains to be further investigated.

Besides the interaction between host gene and gut microbiota, *SYISL* alone plays an important role in the embryonic muscle development. scRNA‐seq technique was employed to analyze the changes in cell composition and myogenic cells in the muscles of WT and KO littermates. As expected, *SYISL* knockout alone significantly increased the number of embryonic muscle fibers as well as the number of myogenic cells and their proportion. These changes were not dependent on HDAC‐H3K9ac/H3K27ac pathway. Interestingly, it is observed the disruption of *SYISL* leads to the blockade of signaling between *MSTN* and its receptors, *tgfbr1* and *Acvr2a*. It is also known that *MSTN*, a member of the TGFβ superfamily,^[^ ^32^ ^]^ inhibits muscle cell proliferation and differentiation,^[^ ^33^ ^]^ as the loss of *MSTN* function could result in increased muscle mass and myofiber number in various species such as mouse, cattle,^[^ ^34^ ^]^ dogs,^[^ ^35^ ^]^ rabbits,^[^ ^36^ ^]^ rats,^[^ ^37^ ^]^ pigs,^[^ ^11^ ^]^ and goats.^[^ ^38^ ^]^ *MSTN* initially binds to the activin type II receptors *Acvr2a* and *Acvr2B*,^[^ ^39^ ^]^ and subsequently to the type I receptors *ALK4* and *tgfbr1* (*ALK5*).^[^ ^40^ ^]^ In mice, the loss of *Acvr2a* leads to a 27–40% increase in muscle mass, while the absence of *tgfbr1* results in an 18% increase.^[^ ^41^ ^]^ Similarly, the knockout of *SYISL* in adult mice results in a 22–31% increase in muscle mass,^[^ ^18^ ^]^ mirroring the phenotypes observed with the absence of *MSTN* and its receptors. However, our scRNA‐seq results showed that knockout of the *SYISL* gene did not cause the significant changes in the mRNA expression of *MSTN* and its receptors, *tgfbr1*, and *Acvr2a*, indicating that *SYISL* knockout may disrupt the signaling pathway between *MSTN* and its receptors through mechanisms beyond mRNA expression, potentially involving post‐translational modifications.

**Figure 10:**
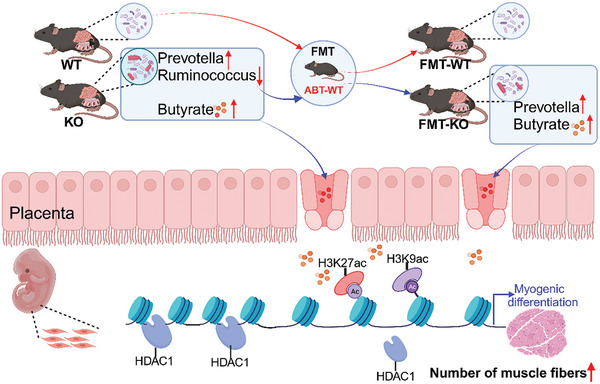
Schematic diagram depicting the molecular mechanism of *SYISL* regulates offspring embryonic muscle fiber number by modulating maternal gut microbiota composition and butyrate levels. *SYISL* KO altered the maternal gut microbiota composition, with both FMT‐KO and KO maternal mice showing a significant increase in the relative abundance of *Prevotella* and serum butyrate levels. Butyrate could crosse the placenta and enter the body of embryonic mice. During embryonic muscle development, butyrate significantly reduces the binding of HDAC1 to myogenic gene regulatory regions, leading to the increased H3K9ac and H3K27ac levels at their regulatory regions. This subsequently elevated gene expression related to myogenesis and contributed to an increase in the number of muscle fibers. Created with BioRender.com.

In summary, *SYISL* knockout significantly altered the composition of the maternal gut microbiota, and increased the abundance of SCFAs‐producing bacteria such as *Prevotella*. These changes led to elevated levels of butyrate in offspring serum, which in turn promoted myogenic differentiation and increased muscle fiber numbers (**Figure** **10**). Meanwhile, the deletion of the *SYISL* gene alone significantly increases the number of embryonic myogenic cells and enhance myogenic differentiation possibly by altering signaling pathways such as *MSTN* and its receptors. These findings illustrate the multifaceted roles of *SYISL* in regulating embryonic muscle development, which advances our understanding of the intricate regulatory network governing muscle development. We believe that the research in this study may act as a strong foundation for developing treatments for muscle‐related diseases and strategies to increase meat production in animal husbandry.

## 4 Experimental Section

### Animals and Treatment

*SYISL* KO mice were generated in our previous study. All WT mice were provided by the Experimental Animal Center of Huazhong Agricultural University. Mating of mice was initiated at 18:00 with a ratio of 1:2 (males: females), and vaginal plugs were checked the next morning at 7:00. The day of vaginal plug detection was considered as embryonic day 0.5. Pseudo germ‐free mice models were established by administering an anhydrous antibiotic cocktail (ampicillin, neomycin, and metronidazole at 0.2 g L^−1^ each, and vancomycin at 0.1 g L^−1^) through gavage for 2 weeks. In the mouse breeding experiments, only KO and ABT‐KO female mice were mated with KO male mice, while the female mice in the other experimental groups (such as fecal microbiota transplantation experiments, butyrate feeding experiments, etc.) were mated with the same male mice as used in the control group. All procedures involving animals strictly adhered to the guidelines of good laboratory practices, with animals provided with nutritious food and ample water. Animal care and experimental protocols were conducted following the guidelines for the care and use of laboratory animals set forth by the National Research Council and approved by the Institutional Animal Care and Use Committee of Huazhong Agricultural University. Approval Number: HZAUMO‐2018‐069.

### Tissue Sectioning and Cell Immunofluorescence Staining

The immunofluorescence staining was performed following previously published methods.^[^ ^42^ ^]^ The immunofluorescence staining antibodies used were MyHC I (Sigma; M8421; 1:500), laminin (Sigma; L9393; 1:400), and MyHC (Santa Cruz Biotechnology; sc‐376157; 1:500). DAPI was used for fluorescence microscopy (DP80; Olympus, Tokyo, Japan). Tissue sections were digitally scanned using a slide scanner (Pannoramic 250FLASH; 3DHISTECH, Hungary). Cell immunofluorescence was performed using confocal laser scanning microscopy (LSM800; Carl Zeiss, Oberkochen, Germany). For each slice sample of EDL muscle, 100 complete muscle fibers from different fields of view were randomly selected for statistical analysis. ImageJ software was used to calculate the area of muscle fibers. Simultaneously, across the entire field of view of the EDL muscle, the same software was used to precisely count the primary (MyHC I), secondary, and total number (laminin) of EDL muscle fibers.

### Western Blotting

Muscle tissue was thoroughly homogenized in a mixture of RIPA buffer and PMSF on ice for 30 min. The homogenate was then centrifuged at 13 000 rpm for 10 min at 4 °C to collect the supernatant containing total proteins. Subsequently, 40 mg of protein was separated on 10% upper and lower gels and transferred onto a polyvinylidene fluoride membrane. Primary antibodies used were anti‐β‐catenin (Santa Cruz Biotechnology; sc‐4777; 1:1000), MyoG (Santa Cruz Biotechnology; sc‐12732; 1:200), MyHC (Santa Cruz Biotechnology; sc‐376157; 1:1000), MYMK (Abclonal; a18158; 1:500), H3K27ac (abcam; ab4729; 1:1000), H3K9ac (abcam; ab32129; 1:1000), and HDAC1 (cell signaling technology; 5356; 1:1000). The secondary antibodies used were horseradish peroxidase‐conjugated anti‐rabbit or anti‐mouse antibodies. Chemiluminescence signals were detected using an ECL detection kit. The intensity of Western blotting images was determined using Image J. β‐actin or histone H3 was used as internal control for data normalization analysis.

### Single Myofiber Isolation and Culture

Single muscle fibers were isolated from the EDL muscle of mice. The muscle was digested with 2 mg mL^−1^ type I collagenase (C0130 Sigma Aldrich, St. Louis, MO, USA) at 37 °C for 1 h. After digestion, the EDL was transferred to preheated Dulbecco's Modified Eagle's Medium (DMEM) containing 10% horse serum, and the muscle was gently flushed using a P200 pipette to release individual muscle fibers. The released single muscle fibers were transferred to a 10 cm DMEM culture dish and cultured in a 37 °C, 5% CO_2_ environment with the addition of 20% fetal bovine serum, 4 ng mL^−1^ basic fibroblast growth factor, and 1% penicillin‐streptomycin.

### rDNA Amplicon Sequencing

DNA extraction from fresh feces collected from various mouse models was performed using a DNA extraction kit to isolate bacterial DNA. Subsequent 16S rRNA amplification and high‐throughput sequencing on the Illumina MiSeq platform were conducted by Wright Labs. Sequences were trimmed at 150 bp, quality filtered at an expected error of less than 0.5% using and analyzed using the QIIME 1.9.0 software package. Downstream chimera removal, alpha diversity, and beta diversity analyses were conducted as described previously.

### Serum SCFAs Detection

After thawing, serum samples were vortexed for 1 min to ensure homogeneity. Then, 50 µL of the sample was transferred to a corresponding 1.5 mL centrifuge tube, followed by the addition of 100 µL of 0.5% phosphoric acid solution. The mixture was vortexed for 3 min. Next, 150 µL of MTBE solvent containing internal standards was added, and the mixture was vortexed for an additional 3 min and sonicated in an ice bath for 5 min to maintain a low temperature. Afterward, the mixture was centrifuged at 12 000 rpm and 4 °C for 10 min. The upper clear supernatant (90 µL) was carefully collected and transferred to a glass‐lined vial for GC‐MS/MS analysis.

### Fecal SCFAs Detection

Approximately 10–30 mg of the fecal samples were homogenized in 500 µL of methanol under 65 Hz conditions. After centrifugation at 12 000 rpm for 15 min at 4 °C, the supernatant was transferred to a 1.5 mL centrifuge tube, and 50 µL of methanol was added for re‐suspension. The previous step was repeated, and finally, 100 µL of the supernatant was subjected to GC 2010 gas chromatography (30.0 m × 0.53 mm CP‐Wax 52CB column) for the detection of short‐chain fatty acid content.

### Feeding Butyrate to Female Mice

The sodium butyrate feeding experiment involved two groups: the sodium butyrate group comprising mice that received a drinking solution of 200 mg of sodium butyrate dissolved in 1 L of distilled water, and the control group, which received plain distilled water. After 7 days on this feeding regimen, the female mice were mated with untreated male mice. The same feeding continued throughout their pregnancy until delivery.

### Isolation and Culture of Skeletal Muscle Satellite Cells

Mouse skeletal muscle satellite cells were isolated from 3‐week‐old C57BL mice. Skeletal muscle satellite cells were isolated and cultured as previously described.^[^ ^43^ ^]^ The muscles were minced and treated with 2 mg mL^−1^ type I collagenase (C0130; Sigma Aldrich, St. Louis, MO, USA). To deactivate the collagenase, digestion was terminated by adding DMEM medium supplemented with 10% fetal bovine serum. The isolated cells were cultured at 37 °C and 5% CO_2_ in a growth medium consisting of RPMI 1640, 20% FBS, 4 ng mL^−1^ basic fibroblast growth factor, 1% chicken embryo extract, and 1% penicillin‐streptomycin, on collagen‐coated cell culture plates.

### Cell Culture

C2C12 cells were grown in incubators at 37 °C and 5% CO_2_, and proliferating cells were cultured in DMEM supplemented with 10% fetal bovine serum (FBS; Gibco, Grand Island, NY, USA). For myogenic differentiation, cells were transferred to DMEM containing 2% horse serum (HS; Gibco). All cells were grown to 80–90% confluence before differentiation was induced.

### RNA Extraction and RT‐qPCR

Total RNA was isolated using TRIzol reagent (Invitrogen, USA) according to the manufacturer's instructions. Total RNA was reverse‐transcribed using Revert Aid Reverse Transcriptase (Thermo Scientific, USA). RT‐qPCR analyses were performed using the Applied Biosystems StepOnePlus real‐time PCR system. Relative RNA expression was calculated using the Ct(2^–∆∆Ct^) method.^[^ ^44^ ^]^ The expression of target genes was normalized to the expression of β‐actin. All primers used in RT‐qPCR are presented in Table S1 (Supporting Information).

### HDACs Activity Assay

For muscle tissue analysis, KO and WT mouse leg muscles were collected, homogenized in liquid nitrogen, and subsequently centrifuged to obtain the supernatant. Blood samples were also centrifuged under similar conditions to obtain the supernatant. Each sample was then diluted in assay buffer at a specified ratio, and aliquots of the diluted samples were added to the wells of a microplate. HDACs enzyme solution, provided as a positive control, and an HDACs inhibitor served as the negative control. 50 µL of HDACs Green substrate working solution were added to each well, and the plates were incubated at a controlled temperature of 35 °C for 30 min. The fluorescence intensity was measured using an appropriate wavelength as specified by the Amplite Fluorimetric HDACs Activity Assay Kit at the end of the incubation period.

### RNA‐Seq Data Analysis

Total RNA was extracted from the leg muscles of 18.5‐day‐old KO and WT mice during the embryonic stage. DNase I treatment was performed to remove genomic DNA from the total RNA. Regarding library construction, as previously reported in the literature.^[^ ^45^ ^]^ The library preparations were sequenced on an Illumina Hi‐seq2000 sequencer and 150 bp paired‐end reads were generated. Raw reads were processed using Trim Galore (v0.6.6) to eliminate low‐quality reads and adapter sequences. Paired‐end reads were then mapped to the mm10 genome using Hisat2 (v2.2.1). To quantify differences in gene expression and obtain raw count data, Feature Counts (v2.0.1) was employed. Subsequently, DEseq2 (v1.28.1) was utilized to normalize the raw counts and perform differential analysis. Genes with |fold change| > 1.5 and a *p*‐value <0.05 were considered significantly differentially expressed,^[^ ^42^ ^]^ and volcano plots and heatmaps were generated for visualization. GO pathway enrichment analysis was performed to gain insights into the functions of the differentially expressed genes.

### Microbial DNA Extraction and Specific Bacteria Quantification

Mice fecal DNA was extracted using the TIANamp Stool DNA Kit (TIANGEN) according to the manufacturer's instructions. Specific bacteria quantitation was measured relative to the universal 16s gene. The primers used for the detection of specific bacteria are listed in Table S1 (Supporting Information).

### ChIP Assay

ChIP assays were performed after transfection of butyrate into mouse skeletal muscle satellite cells using the ChIP Assay Kit (Beyotime, Jiangsu, China). Each ChIP assay was performed using 1 µg of antibodies against H3K27ac (ab4729, abcam, 1:100), H3K9ac (ab32129, abcam, 1:100). IgG was used as the negative control. RT‐qPCR was conducted using the retrieved DNA and the primers for ChIP to check the enrichments of H3K9ac, and H3K27ac at target gene regions. All primers used in ChIP‐qPCR are presented in Table S1 (Supporting Information).

### Preparation of Single‐Cell Suspension

Heterozygous female mice at 18.5 days of gestation were used for fetal sampling. The leg muscles of each fetus were individually isolated and placed in a single‐cell preservation solution. Fetal tails were used for genotyping. Upon successful identification, the leg muscle tissues from both WT and KO mice were digested using 0.25% trypsin (Thermo Fisher; 25200‐072) and 10 ug mL^−1^ DNase I (Sigma, 11284932001) dissolved in PBS containing 5% FBS (Thermo Fisher; SV30087.02). The digestion was carried out at 37 °C on a shaker for 40 min. The resulting cell suspensions were filtered through a 40 µm nylon cell strainer. Subsequently, cells were stained with 0.4% Trypan blue (Thermo Fisher, 14190144) to assess viability using the Countess II Automated Cell Counter (Thermo Fisher).

### 4.1 Library Preparation and Sequencing

Beads with unique molecular identifiers (UMIs) and cell barcodes were filled in Gel Beads‐in‐emulsion (GEM), pairing each cell with a bead. After cell lysis treatment, cell RNA hybridized to the beads, which were then processed for reverse transcription. During cDNA synthesis, each molecule received a UMI and cell label tag at the 5' end. Subsequently, the beads went through second‐strand synthesis, adaptor ligation, and amplification. Sequencing libraries, focusing on the 3' transcript ends with cell barcodes and UMIs, were prepared according to the Chromium Single Cell 3ʹ v3 protocol. Library quality and quantity were assessed using a Bioanalyzer 2100 and a Qubit assay. Sequencing was performed on an Illumina NovaSeq6000 using 2 × 150.

### scRNA‐Seq and Data Pre‐Processing

scRNA‐seq data were processed using the CellRanger toolkit (v7.2.0). Raw sequencing reads were aligned to the mouse reference genome (mm10) to generate data matrices. High‐quality single‐cell data selection for downstream analysis was based on the following criteria:

1. Gene detection count: Cells with gene counts ranging from 1000 to 6000 were retained. This range was chosen to exclude cells with abnormally low or high expression levels, as well as potential doublets.
2. Total RNA count: Only cells with total RNA counts between 1000 and 25 000 were selected, minimizing the inclusion of cells with degraded RNA.
3. Mitochondrial gene expression percentage: Cells where less than 8% of the total gene expression was mitochondrial were chosen, reducing the likelihood of including damaged cells.

### Data Normalization and Batch Correction

For WT and KO samples, normalization was conducted using the variance stabilizing transformation (VST) method via the Seurat package (v5.1.0). A total of 3000 highly variable genes were identified and conducted PCA for dimensionality reduction. The Harmony package (v1.2.0) was utilized to address batch effects across samples processed under similar experimental conditions. After correcting for batch effects, WT and KO samples were combined using the merge function within the Seurat package. Following this integration, the combined dataset was normalized using the ScaleData function.

### Clustering and Cell Annotation

The analysis continued with the application of the FindNeighbors function, set at a resolution of 0.3. This was followed by clustering using the FindClusters function and visualization through t‐distributed stochastic neighbor embedding (t‐SNE) via the RunTSNE function. Following data preprocessing, differential gene expression analysis was performed to identify genes significantly differing across clusters. This was accomplished using the FindAllMarkers function within the Seurat package. The selection parameters for DEGs were a log fold change > 0.25 and a *p*‐value < 0.05. Subsequently, cell populations were annotated by correlating these DEGs with well‐documented cellular markers from literature, facilitating precise cellular identification and characterization.

### Single‐Cell Trajectory Analysis

To explore the heterogeneity and developmental trajectories of myogenic cells, Monocle 2 (version 2.30.1) was utilized for pseudotime trajectory analysis of scRNA‐seq data. Initially, count data were extracted from the Seurat object using the GetAssayData function, which were then converted into a CellDataSet object. During this conversion, a negative binomial model was employed to more accurately capture the discrete nature of gene expression. Subsequently, dimensionality reduction was applied using the DDRTree algorithm. After reducing dimensionality, cells were temporally ordered using the order cells function, which established their relative positions within the differentiation process. Additionally, key pseudotime genes with adjusted *p*‐values less than 1e‐4 were identified, whose expression patterns were essential for understanding shifts in cell states.

### Expression Heatmap Plotting and Gene Function Analysis

Using the plot_pseudotime_heatmap function from the Monocle package, expression heatmap of these key genes was plotted across different cell states, visually displaying the dynamic changes in gene expression. Based on expression patterns, genes were further clustered into four groups using the k‐means clustering method. The function of each gene cluster was investigated through GO analysis accessed via the DAVID database (https://david.ncifcrf.gov/).

### Cell‐Cell Communication Analysis

To quantitatively assess differences in intercellular communication between KO and WT samples, the R package CellChat (v1.6.1) was utilized. The netVisual_diffInteraction function was used to generate visual representations of the differential interaction networks between two sample groups. Subsequent analysis was conducted using the rankNet function to identify the primary signaling pathways that exhibited significant changes. Further detailed visualization and examination of specific pathways were facilitated through the netVisual_aggregate and netAnalysis_signalingRole_network functions, allowing for an in‐depth exploration of the altered signaling dynamics.

### Statistical Analyses

All data were presented as mean ± standard deviation (SD). Sample sizes (*n*) were indicated in the legends. Statistical analysis was performed using GraphPad Prism software. For comparisons between two groups, both paired and unpaired Student's *t*‐tests were employed. For analyses involving three or more groups, R software (version 4.1) was utilized to conduct multiple comparison statistical analyses. An initial analysis of variance (ANOVA) was carried out using the aov function to assess significant differences across group means. This was followed by pairwise comparisons using the Tukey HSD function to pinpoint specific groups among which the differences were statistically significant. *p* < 0.05 was considered to be statistically significant; significance was denoted as *p < 0.05, **p < 0.01, ***p < 0.001.

## Conflict of Interest

The authors declare no conflict of interest.

## Author Contributions

H.Z. and W.J. wish it to be known that, in their opinion, the first two authors should be regarded as joint first authors. H.Z. and W.J. contributed equally to this work. H.Z., W.J., J.G., C. L., Y.P., J.J., W, L., X. L., J. H., M.Z., Y.J., Z.X., J.T., R.Z., and B.Z. conceived and designed the research; H.Z., J.G., C.L, Y.P., X.L., J.H. performed experiments; W.J., Z.M., and X.Z. analyzed the data; H.Z., W.J., J.G., R.Z., and B.Z. wrote the manuscript. All authors read and approved the final manuscript.

## Supporting information



Supporting Information

## Data Availability

The data that support the findings of this study are openly available in [Gene Expression Omnibus (GEO)] at [https://www.ncbi.nlm.nih.gov/geo/query/acc.cgi?acc=GSE244822, https://www.ncbi.nlm.nih.gov/geo/query/acc.cgi?acc=GSE276532], reference number [GSE244822 and GSE276532].
