# Supplementary material for: SYISL Knockout Promotes Embryonic Muscle Development of Offspring by Modulating Maternal Gut Microbiota and Fetal Myogenic Cell Dynamics
Source: Adv Sci (Weinh). 2024 Dec 16;12(6):2410953. doi: 10.1002/advs.202410953 (PMC11809340; doi:10.1002/advs.202410953)

**Supporting Information**

***SYISL* Knockout Promotes Embryonic Muscle Development of Offspring by Modulating Maternal Gut Microbiota and Fetal Myogenic Cell Dynamics**

*Hao Zuo*^#^, *Wei Jiang*^#^, *Jianwei Gao*, *Zhibo Ma*, *Chen Li*, *Yaxin Peng*, *Jianjun Jin*, *Xizhen Zhan*, *Wei Lv*, *Xiao Liu*, *Jingjing Hu*, *Mengdi Zhang*, *Yiming Jia*, *Zaiyan Xu*, *Junming Tang*, *Rong Zheng*^*^, *and* *Bo Zuo*^*^


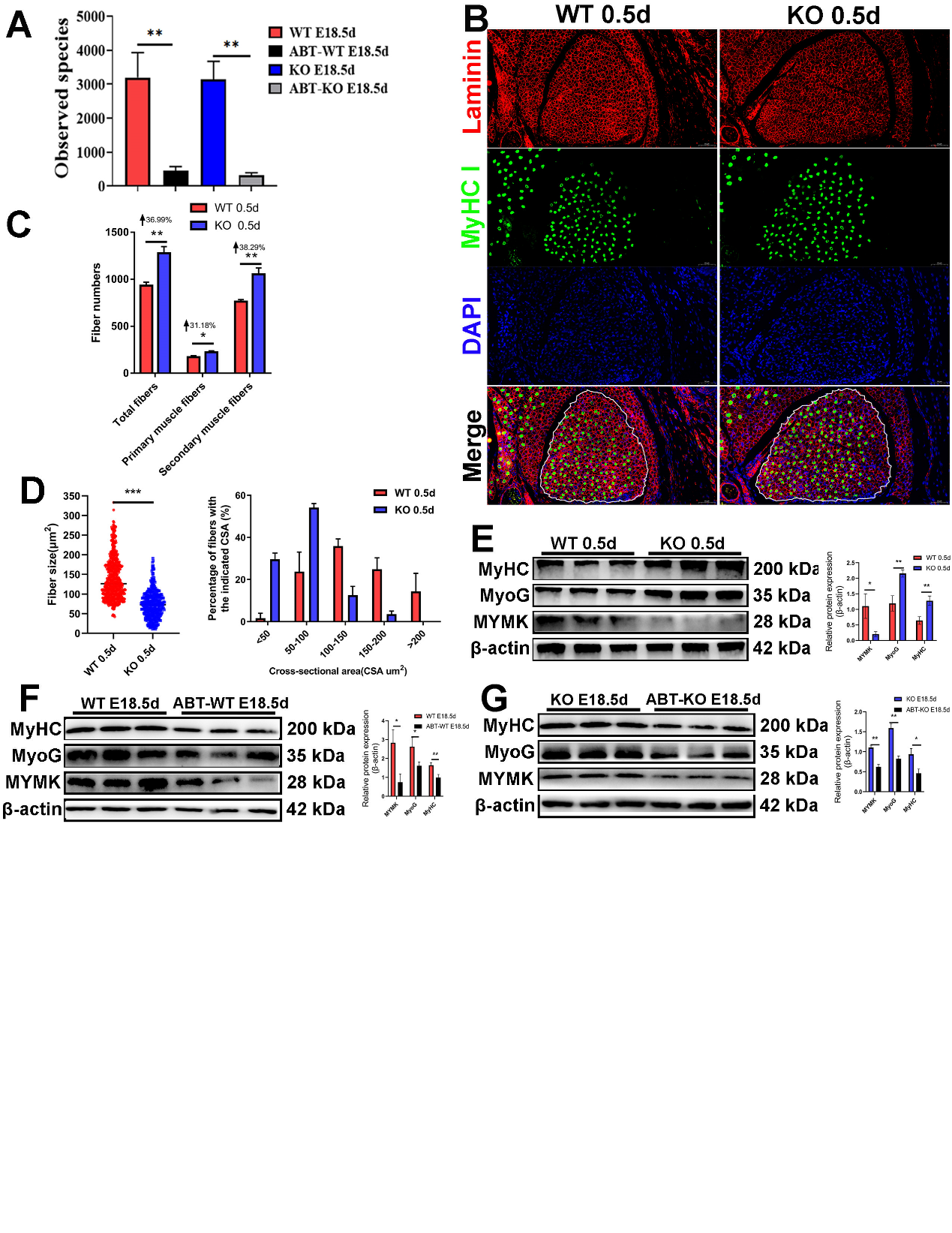


**Figure S1. The muscle fiber phenotype of KO and WT mice at postnatal day 0.5.** A) α diversity analysis was performed to confirm the successful construction of pseudo-germ-free mice. B) Representative images of immunofluorescence staining for laminin (red), MyHC I (green), and DAPI (blue) in the cross-sections of the hind limbs of KO and WT mice at 0.5d. The white dashed lines delineate the mouse EDL muscle. (n = 6/each group) Scale bars, 50 μm. n = 5. C) Quantitative analysis of immunofluorescence staining was conducted on the leg muscles of mice at 0.5d post-birth, and the results revealed a significant increase in the number of primary, secondary, and total muscle fibers in the KO group compared to the control group. Data were presented as mean ± SDs, n = 3. **P* < 0.05, ***P* < 0.01. D) The cross-sectional areas of individual EDL muscle fibers at 0.5d were analyzed. The left figure presents a scatter plot of the areas of each muscle fiber, and the right figure shows the distribution of the muscle fibers with different area ranges. Compared to WT mice, KO mice exhibit a higher proportion of muscle fibers with smaller cross-sectional areas. Data were presented as mean ± SDs, n = 3. **P* < 0.05, ***P* < 0.01. E) Western blotting results showed that *SYISL* KO significantly increased the protein expression levels of MyoG and MyHC genes and notably decreased the protein expression levels of MYMK genes at 0.5d. Data were presented as mean ± SDs, n = 3. **P* < 0.05, ***P* < 0.01. F, G) Western blotting results showed at E18.5 d, ABT-WT (F) and ABT-KO (G) significantly decreased the protein expression levels of the MYMK, MyoG and MyHC genes compared to the control group. The relative protein and gene levels were normalized to those of the control β-actin. Data were presented as mean ± SDs, n = 3. **P* < 0.05, ***P* < 0.01.


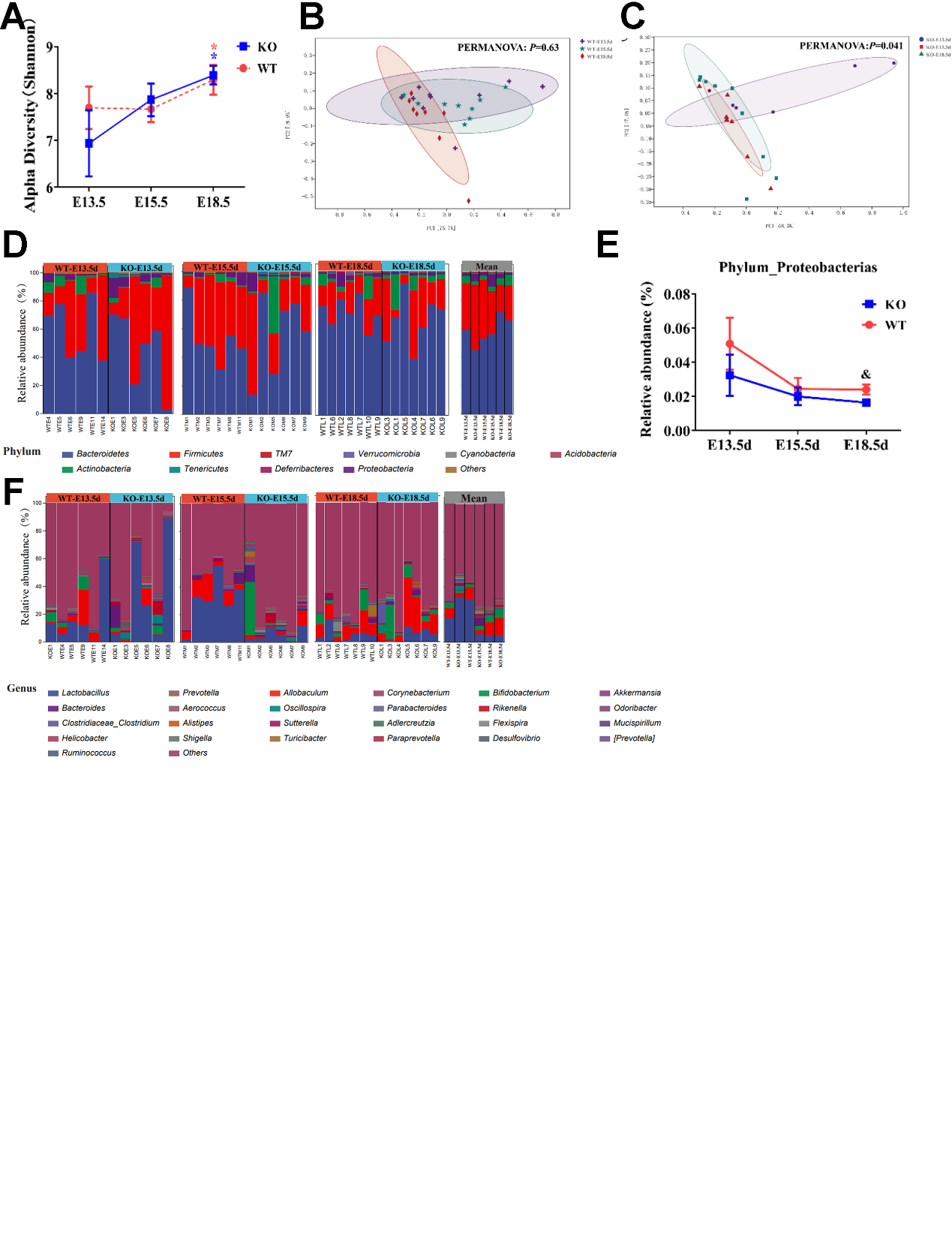


**Figure S2. *SYISL* knockout in female mice significantly affects the gut microbiota composition.** A) The analysis of the Shannon index reflects the changes in gut microbiota alpha diversity in WT and KO female mice at different stages of pregnancy. Both genotypes of mice showed an increasing trend in alpha diversity, with a significant increase at E18.5d compared to E13.5d. The highest species richness was observed in female mice at E18.5d. B,C) PCoA analysis of fecal microbiota in WT (B) and KO (C) female mice during all three stages of pregnancy revealed that there was no significant variation in the WT group as pregnancy progressed, while the KO group exhibited significant differences. D, F) A bar chart depicting the species composition at the phylum (D) and genus (F) levels of fecal microbiota in WT and KO female mice during all three stages of pregnancy. E) The relative abundance changes of fecal microbiota in WT and KO female mice during all three stages of pregnancy for microbial groups phylum *Proteobacteria*. The data represent the means ± SDs of three independent experiments; * *P* < 0.05, ** *P* < 0.01.


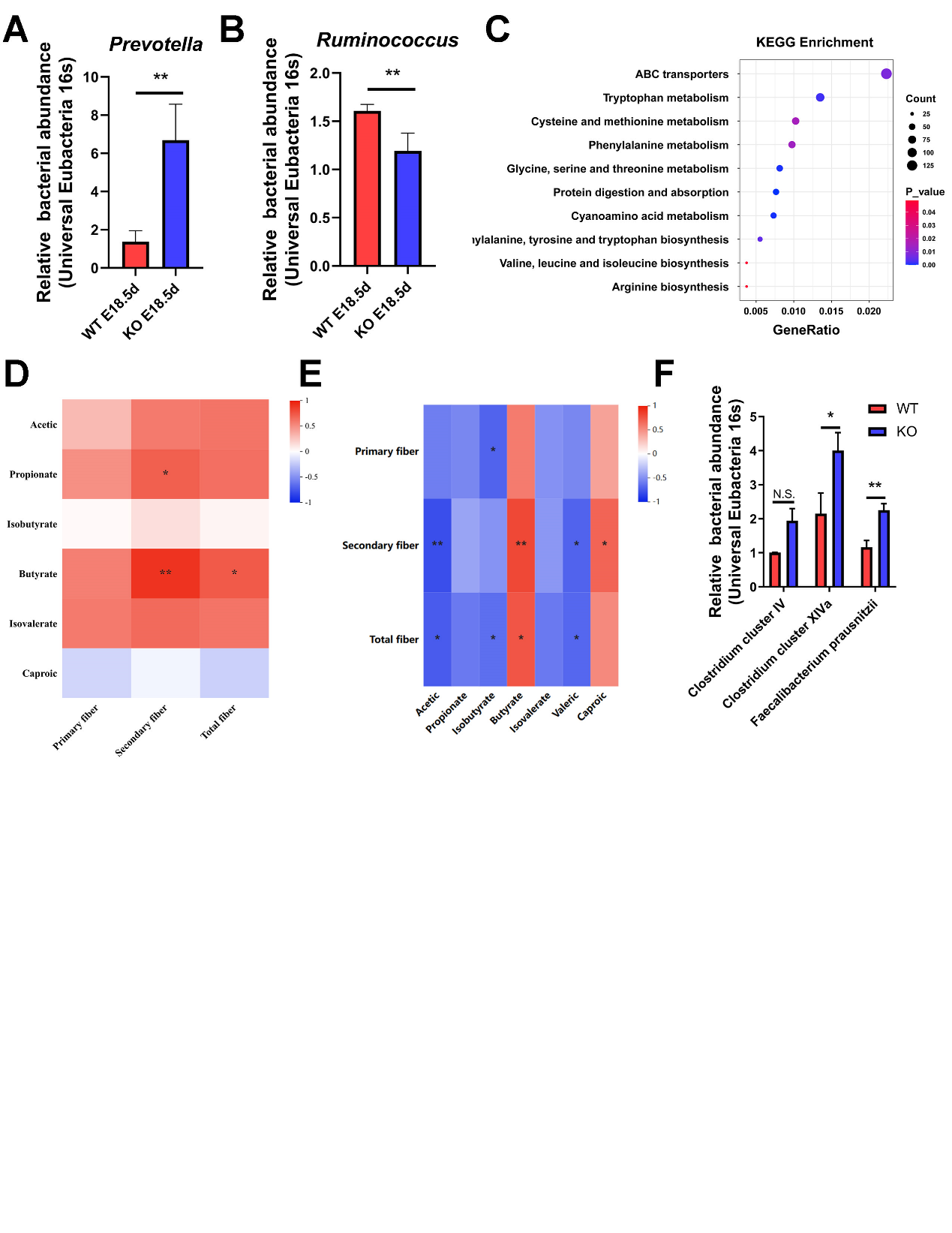


**Figure S3. Fecal bacterial analysis and metabolite pathway analysis.** A, B) The RT-qPCR results showed that in pregnant mice at 18.5 days, compared to the control group, KO *SYISL* significantly increased the abundance of *Prevolla* (A), while *Ruminococcus* (B) significantly decreased in the fecal samples of maternal mice when universal Eubacteria 16S was used as the reference gene. Data were presented as mean ± SDs, n = 3. ***P* < 0.01. C) KEGG pathway analysis of fecal metabolites from 18.5-day-old pregnant WT and KO female mice reveals significant alterations in biological pathways associated with protein degradation, transport, and amino acid metabolism resulting from gene knockout. D, E) Heatmap representation of the correlation analysis between SCFAs in maternal feces (D) and serum (E) and the number of embryonic muscle fibers. F) The RT-qPCR results showed that KO *SYISL* significantly increased the abundance of the butyrate-producing bacteria *Clostridium cluster XIVa* and *Faecalibacterium prausnitzii* in the fecal samples of maternal mice when universal Eubacteria 16S was used as the reference gene. Data were presented as mean ± SDs, n = 3. N.S. not significant, **P* < 0.05, ***P* < 0.01.


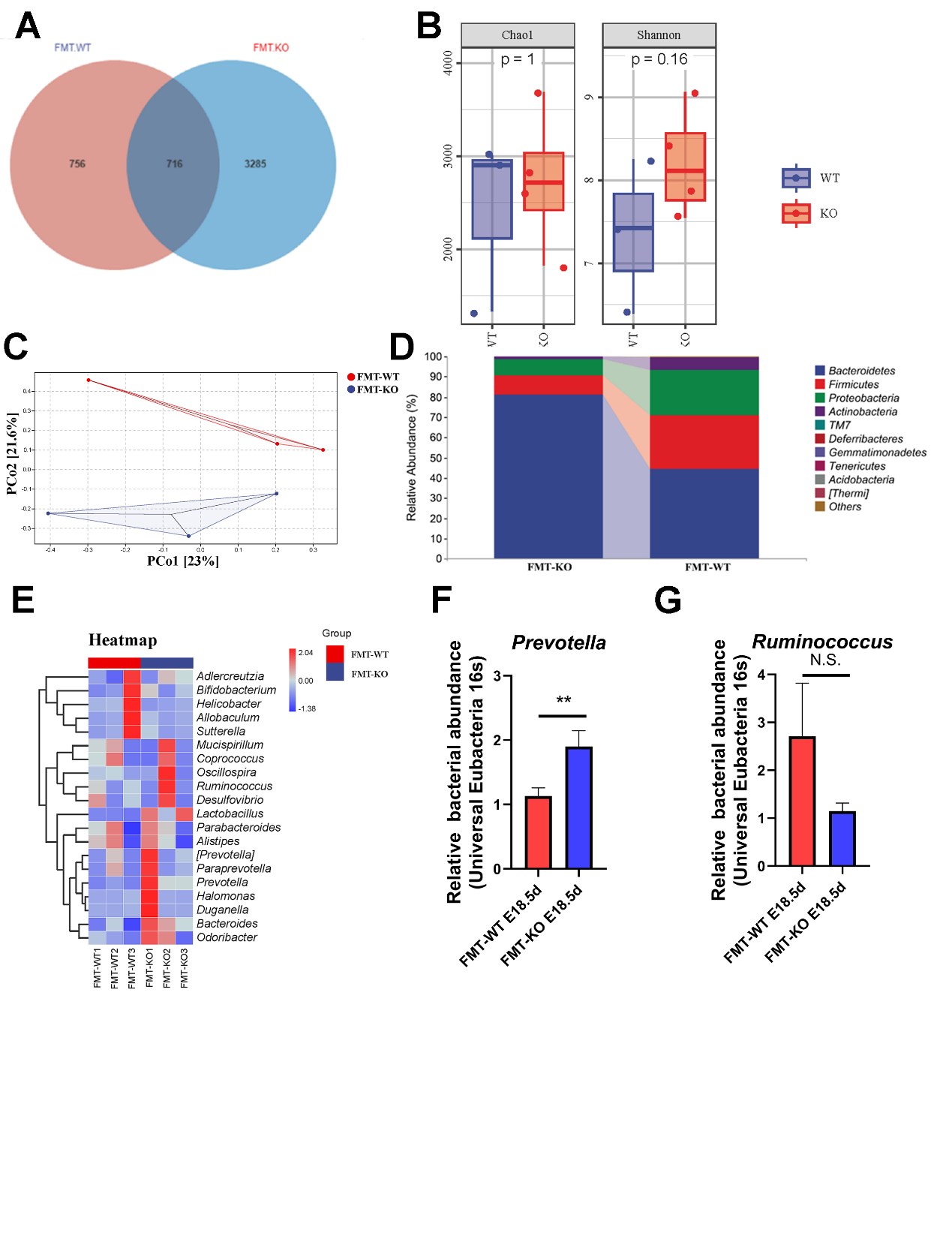


**Figure S4. FMT of KO fecal microbiota in ABT mice results in more muscle fibers in offspring than that of WT fecal microbiota.** A) Distribution of FMT-WT and FMT-KO gut microbiota based on OTU analysis. Microbiota profiling identified a total of 4715 OTUs, with 716 OTUs shared by both groups, 756 OTUs specific to FMT-WT pregnant mice, and 3285 OTUs specific to FMT-KO pregnant mice. n = 3. B) The Chao1 and Shannon indices indicate that there was no difference in microbial diversity between FMT-KO and FMT-WT mice at gestational day 18.5. C) PCoA results of gut microbiota indicate differences in the gut microbial communities between FMT-WT and FMT-KO mice at gestational day 18.5. n = 3. D) Column chart depicting that fecal there was significant difference in microbial composition at the phylum level between FMT-WT and FMT-KO mice at gestational day 18.5. E) The heatmap depicting fecal microbiota composition at the genus level between FMT-WT and FMT-KO mice at gestational day 18.5 shows a significant increase in the abundance of *Prevotella* in FMT-KO mice compared to FMT-WT mice. n = 3. F, G) The RT-qPCR results showed that in pregnant mice at 18.5 days, compared to the control group, FMT-KO significantly increased the abundance of *Prevolla* (F), while *Ruminococcus* (G) significantly decreased in the fecal samples of maternal mice when universal Eubacteria 16S was used as the reference gene. Data were presented as mean ± SDs, n = 3. N.S. not significant, ***P* < 0.01.


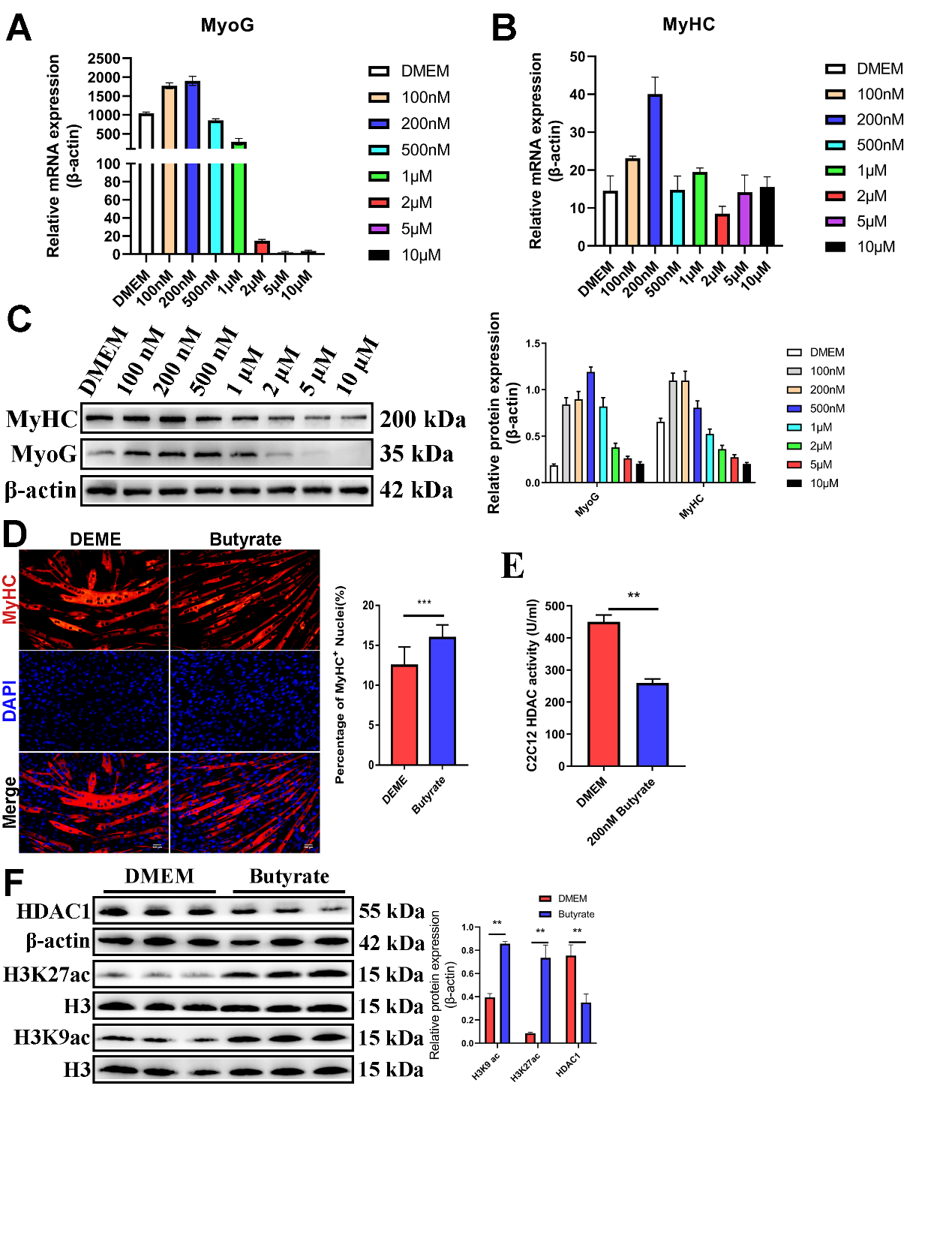


**Figure S5. 200nM butyrate significantly promotes myogenic differentiation, suppresses the activity of histone deacetylases (HDACs) and increases H3K9ac and H3K27ac levels in C2C12 cells.** A, B) The RT-qPCR results revealed that when cells were treated with different concentrations of butyrate, 100-200 nM butyrate promoted the expression of *MyoG* (A) and *MyHC* (B) genes when *β-actin* was used as the reference gene. C) Western blotting results revealed that when cells were treated with different concentrations of butyrate, 100-500 nM butyrate promoted the expression of *MyoG* and *MyHC* genes when *β-actin* was used as the reference gene. D) Representative images of immunofluorescence staining for MyHC in differentiated C2C12 myoblasts and quantification showed that butyrate significantly promoted myoblast differentiation. Scale bars, 100 μm. Data were presented as mean ± SDs, n = 3. ****P* < 0.001. E) C2C12 cells were treated with different concentrations of butyrate to induce differentiation, and the supernatant was subjected to an HDAC enzyme activity assay. The results showed there was a significant reduction in HDAC enzyme activity in C2C12 cells treated with butyrate. Data were presented as mean ± SDs, n = 3. ***P* < 0.01. F) Western blotting results showed that treatment of C2C12 cells with 200 nM butyrate for 3 d inhibits HDAC1 protein expression and increases the protein levels of H3K27ac and H3K9ac when β-actin and H3 were used as the reference gene. Data were presented as mean ± SDs, n = 3. ***P* < 0.01.


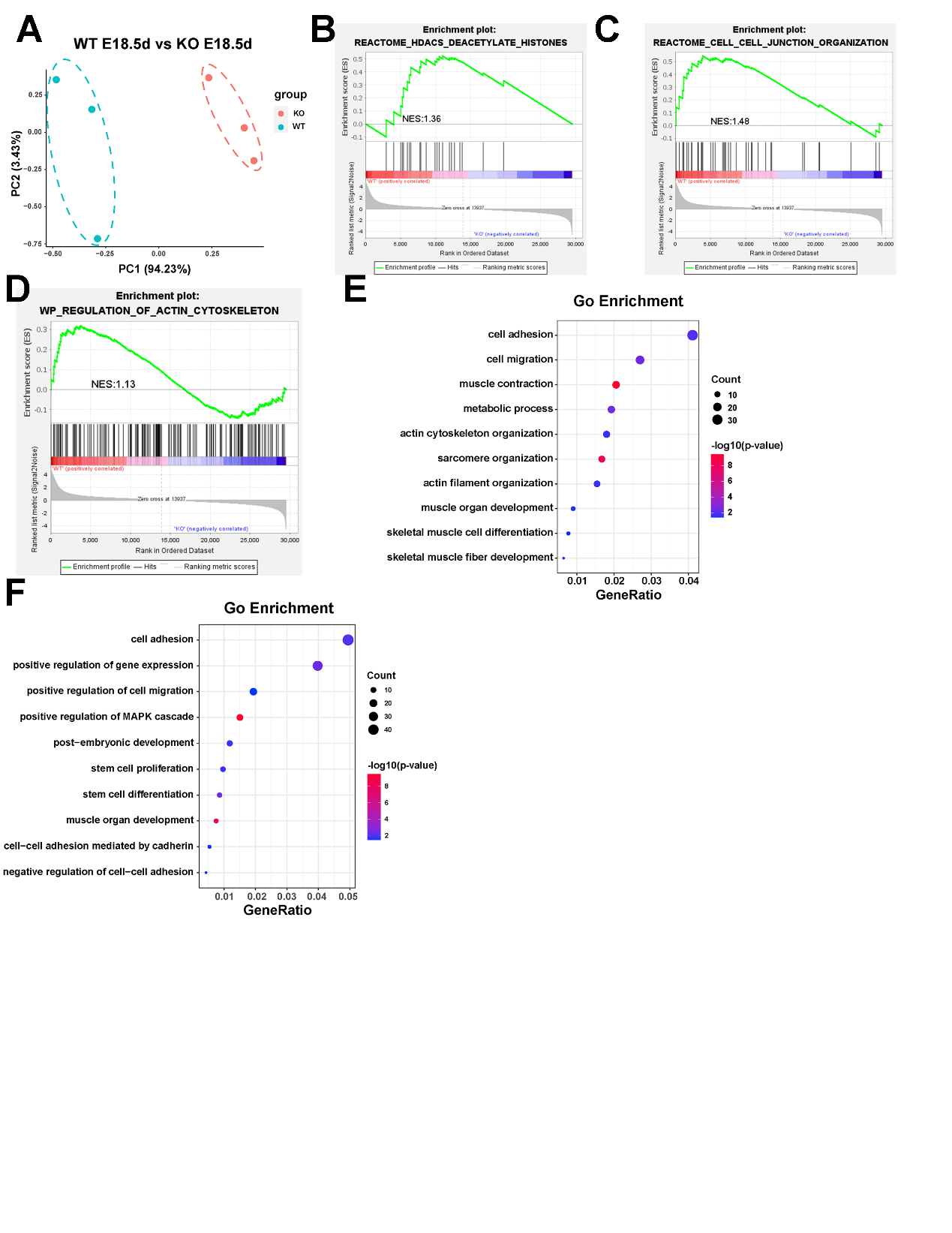


**Figure S6. GSEA and GO Enrichment analysis of DEGs.** A) Analysis of RNA-seq data from KO and WT mouse leg muscles at E18.5d showed there was distinct separation between the two groups in the PCA. n = 3. B-D) GSEA plot of DEGs related to HDACs deacetylate histones (B), cell-cell junction organization (C), regulation of actin cytoskeleton (D) in leg muscle from WT mice and KO mice at E18.5d. E) GO Enrichment analysis of 259 overlapping genes between RNA-seq and H3K9ac ChIP-seq data indicated these genes were mainly related to cell adhesion, cell migration, muscle differentiation, and metabolic pathways. F) GO enrichment analysis of 934 overlapping genes between RNA-seq and H3K27ac ChIP-seq data indicated these genes were mainly related to cell adhesion, stem cell differentiation and muscle organ development.


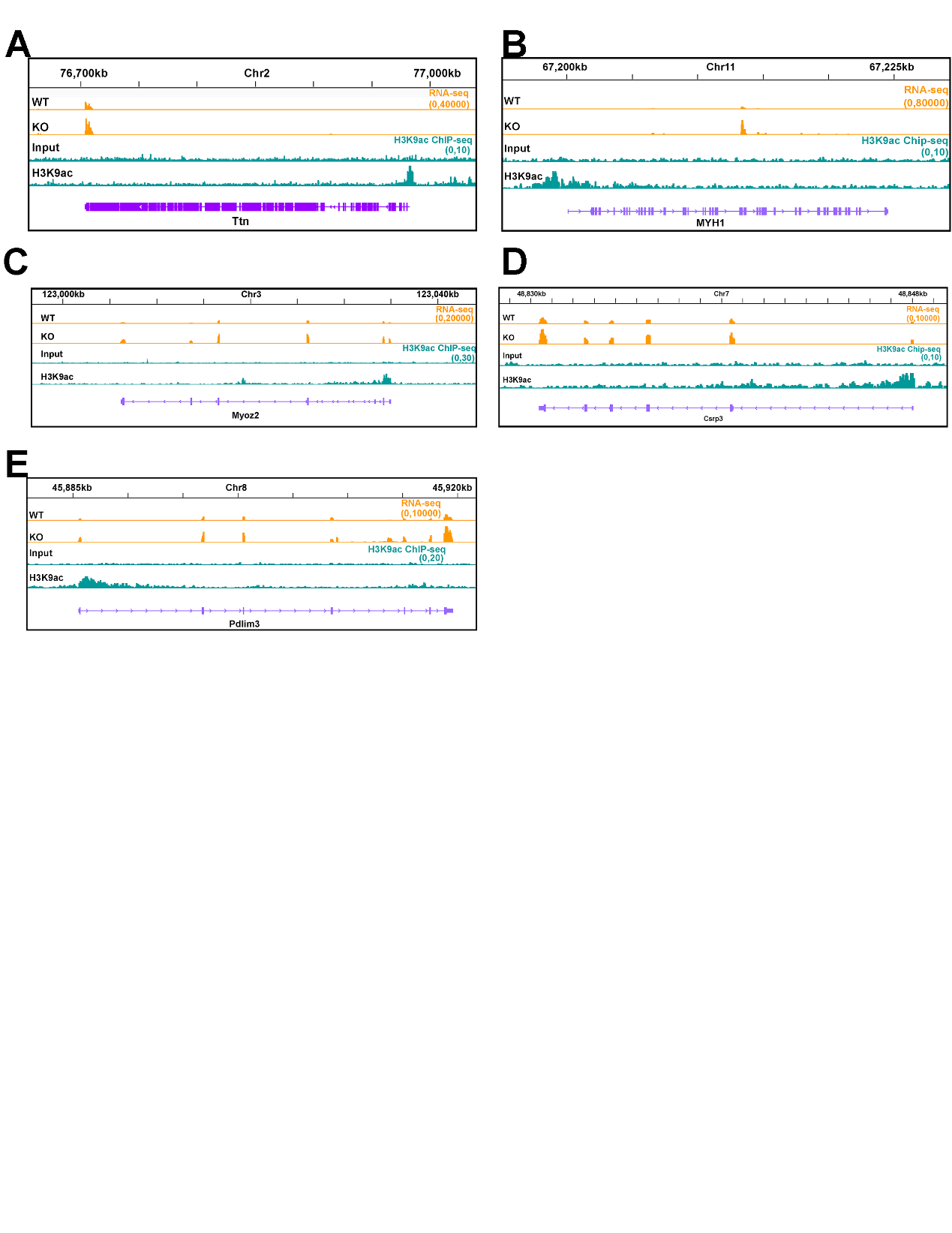


**Figure S7. Integrated RNA-seq and ChIP-seq profiles for gene expression and epigenetic markers.** A-E) Combined profiles of RNA-seq (orange), H3K9ac (green) ChIP-seq profiles for *Ttn (A), Myh1 (B), Myoz2(C), Csrp3(D), Pdlim3(E).* The RNA-seq results showed a significant increase in *Ttn (A), Myh1 (B), Myoz2(C), Csrp3(D), Pdlim3(E)* expression after *SYISL* knockout, and the H3K9ac ChIP-seq results showed that there were H3K9ac enrichments at their regulatory region.


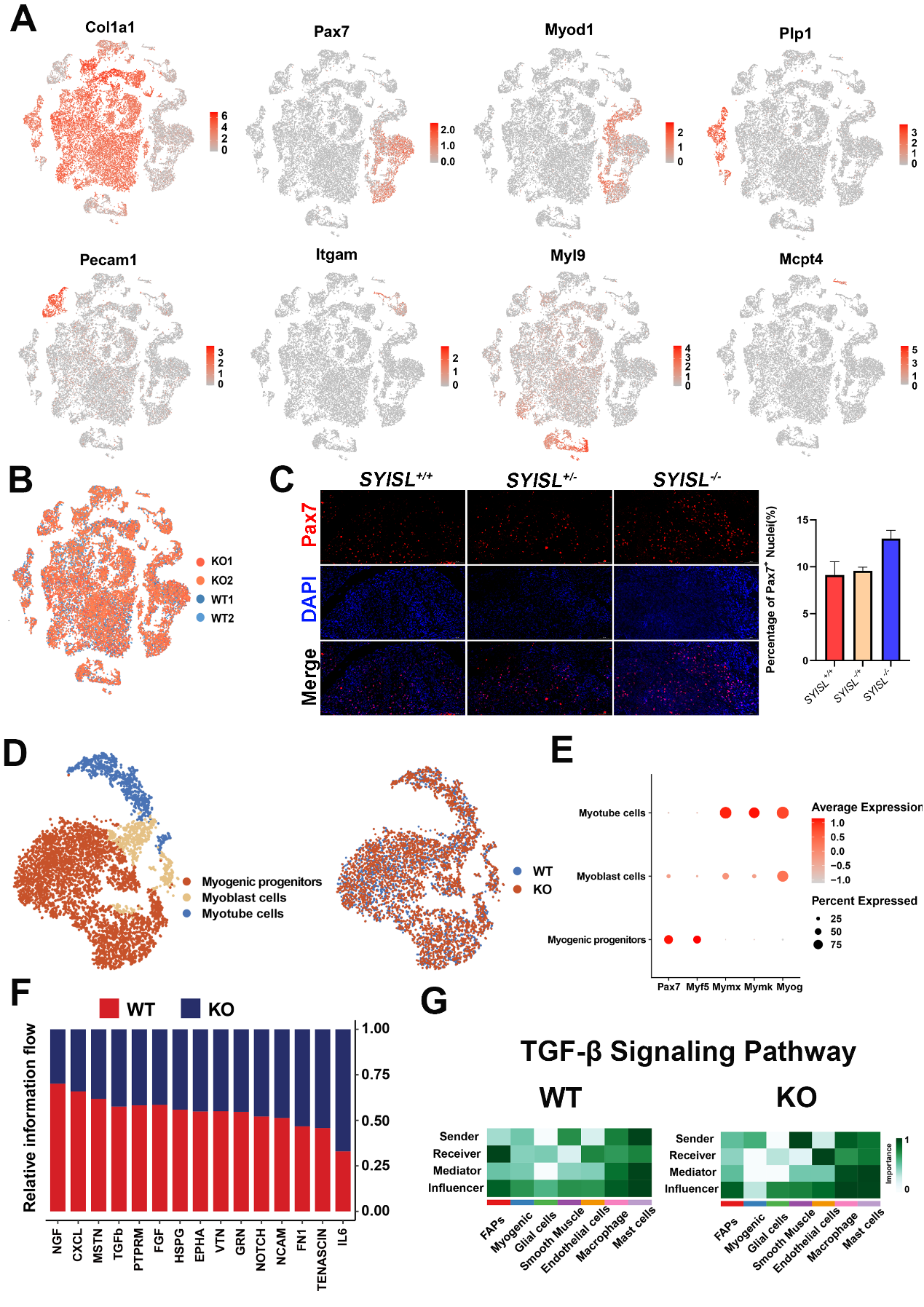


**Figure S8.** **Single-cell analysis of leg muscles from littermate mice with different genotypes.** A) The t-SNE plots depict the expression of marker genes of transcriptional profiling of leg muscles, facilitating the identification of distinct cellular clusters. The t-SNE axes reveal the distribution of cells, while the gradient of color from gray to red illustrates the relative expression levels of each gene. Quantitative values are indicated on the color scale bars located on the right side of each plot. B) t-SNE plot representing the single-cell profiles of leg muscles from various samples, with colors identifying distinct samples. C) Representative images of immunofluorescence staining for Pax7 in both *SYISL*^+/+^, *SYISL*^-/+^and *SYISL*^-/-^ fetuses at E18.5d and quantification analysis of immunofluorescence results showed that compared with WT (*SYISL*^+/+^) fetuses, Pax7 protein expression in muscles was significantly upregulated *SYISL*^+/-^ and *SYISL*^-/-^ fetuses. Scale bars, 100 μm. D) Two different t-SNE plots are used to visualize the various types of muscle cells (left plot) and their genotypic classifications (right plot). The cell types are primarily divided into Myogenic progenitors (red), Myoblast cells (yellow), and Myotube cells (blue); genotypes are classified into WT (blue) and KO (red). E) This dot plot illustrates the expression levels of marker genes across different stages of myogenesis, including Myogenic progenitors (*Pax7^+^/Myf5^+^*), Myoblast cells (*Myog^+^/Mymk^-^*), and Myotube cells (*Mymx^+^/Mymk*^-^). The vertical axis lists the different subgroups, and the horizontal axis categorizes the marker genes based on their expression in these cell types. The size of the dots represents the proportion of cells expressing each gene, while the color intensity indicates the relative levels of gene expression. F) Bar graph illustrating differential signaling pathways between WT and KO samples within all cells. Each bar represents the relative information flow through various pathways, with red bars indicating WT and blue bars representing KO. G) Communication networks of TGF-β pathways analyzed using CellChat. The heatmap displays the roles of each cell type in the communication network. The intensity of the color in the heatmap corresponds to the level of activity or influence of each cell type within these roles.

Table S1. Sequence of primers for RT-qPCR and ChIP-qPCR

| Name | Sequence（5’-3’） | Application |
| --- | --- | --- |
| *SYISL* | F: CTCGTGGTCCCTCCCTGTAA | RT-qPCR |
|  | R: GTCTGCGTGCTCCTGTGGTT |  |
| *MyoD* | F: CGAGCACTACAGTTGGCGACTAAGA | RT-qPCR |
|  | R: GCTCCACTATGCTGGACAGGCAGT |  |
| *Myoz2* | F: TTAAGATGCGACAAAGAAGATC | RT-qPCR |
|  | R: GTTAGGAGGAGTAAATGGTGCT |  |
| *Myh2* | F: AGTCCCAGGTCAACAAGCTG | RT-qPCR |
|  | R: GCATGACCAAAGGTTTCACA |  |
| *Myh7* | F: AGTCCCAGGTCAACAAGCTG | RT-qPCR |
|  | R: TTCCACCTAAAGGGCTGTTG |  |
| *Myh4* | F: GAGTTCATTGACTTCGGGATGG | RT-qPCR |
|  | R: TGCTGCTCATACAGCTTGTTCTTG |  |
| *Myh1* | F: AAGGGTCTGCGCAAACATGA | RT-qPCR |
|  | R: TTGGCCAGGTTGACATTGGA |  |
| *Lama2* | F: TGCTGTCCTGAATCTTGCTTC | RT-qPCR |
|  | R: AGCATTTGTAATCGGGTGTCTC |  |
| *Tet2* | F: CACCCATCCACACCCTTCACC | RT-qPCR |
|  | R: TCTGGAGGCAGCTCCCATGAA |  |
| *Trim72* | F: TCCCTGTTGTCAGGCATCTA | RT-qPCR |
|  | R: TTCTTCCACACCTGGAATTT |  |
| *eMyhc* | F: TATCAGAGTGAGGAGGACAG | RT-qPCR |
|  | R: TCGCTTTCATGGACCACCAT |  |
| *Cavin4* | F: ACAGTCACACAGCAATACGGGCTA | RT-qPCR |
|  | R: TTCTCGGGCAGGCTTCTGTCTTTA |  |
| *Cldn23* | F: ATGGATGTGCTTGAGGGAGAAG | RT-qPCR |
|  | R: TCACAGGGCAGCGAATTTTG |  |
| *Kirrel3* | F: ACTGCACACCCAAGTTGCCCG | RT-qPCR |
|  | R: TCAGGTGATGCTCTCCTGAGAG |  |
| *Celsr3* | F: GCAGTCCTCAAGCACTCCCTC | RT-qPCR |
|  | R: CCTGGTGGCTCCTCATTCG |  |
| *Myh13* | F: CCCATCCTCACTTTGTGC | RT-qPCR |
|  | R: CGAATCGGAACTGCTCT |  |
| *TMEM182* | F: TCAGTGGCGTTGTGGA | RT-qPCR |
|  | R: GGAGTCGTAGGAGGTGG |  |
| *Cfl2* | F: CCGACCCCTCCTTCTTCTCG | RT-qPCR |
|  | R: GTAACTCCAGATGCCATAGTG |  |
| *Trim63* | F: ACGAGAAGAAGAGCGAGCTG | RT-qPCR |
|  | R: CTTGGCACTTGAGAGGAAGG |  |
| *Actn2* | F: TGATCCAGAGCTACAGCATCCG | RT-qPCR |
|  | R: CAGACGCTCATTAGCATGTTGG |  |
| *Aldoa* | F: ACATTGCTGAAGCCCAACAT | RT-qPCR |
|  | R: ACAGGAAAGTGACCCCAGTG |  |
| *Pdlim3* | F: TCCCCGGATCACTGCAAAAT | RT-qPCR |
|  | R: GTCATCAGGCCCGTCATTCA |  |
| *Pdlim5* | F: TGACAGAATCTGAAAATGACAA | RT-qPCR |
|  | R: TGGTTTGGACGCTGCCAGCTAGG |  |
| *CSRP3* | F: TGTAAGGTGTGCTATGGGCG | RT-qPCR |
|  | R: TTGGAAGGGTTGCTTGTGGT |  |
| *β-actin* | F: GCCTCACTGTCCACCTTCCA | RT-qPCR |
|  | R: AGCCATGCCAATGTTGTCTCTT |  |
| *Prevotella* | F: CACCAAGGCGACGATCA | RT-qPCR |
|  | R: GGATAACGCCTGGACCT |  |
| *Ruminococcus* | F: GGACTGCATTTGGAACTGTCAG | RT-qPCR |
|  | R: AACGTCAGTCATCGTCCAGAAAG |  |
| *Clostridium cluster XIVa* | F: AAATGACGGTACCTGACTAA | RT-qPCR |
|  | R: CTTTGAGTTTCATTCTTGCGAA |  |
| *Clostridium cluster IV* | F: GCACAAGCAGTGGAGT | RT-qPCR |
|  | R: CTTCCTCCGTTTTGTCAA |  |
| *Faecalibacterium prausnitzii* | F: CCCTTCAGTGCCGCAGT | RT-qPCR |
|  | R: GTCGCAGGATGTCAAGAC |  |
| *Csrp3* | F: ATGAACTCCTATTTACGTCCT | ChIP-qPCR |
|  | R: CCTCACATCAAAAGAGTGCAA | (H3K9ac) |
| *Myoz2* | F: GGCACGTTTATAGTAATTGCTT | ChIP-qPCR |
|  | R: CTTGGATTTGTAACGAGCTTC | (H3K9ac) |
| *Myh1* | F: AGGACCAGCACCACATATGAAG | ChIP-qPCR |
|  | R: CTCCTGAGAACAGACAAGCG | (H3K9ac) |
| *Ttn* | F: CAACCAAGGCAGCATGGAAC | ChIP-qPCR |
|  | R: AAAACATAATGTACGCCTTT | (H3K9ac) |
| *Tet2* | F: CGACAAGGGAGCTAAAACCA | ChIP-qPCR |
|  | R: TCGCATTCTTCTACACGA | (H3K9ac) |
| *Cfl2* | F: AGTGGCCTCCTACCCCAAC | ChIP-qPCR |
|  | R: TTTCTCCCCACCTTAGGGTCT | (H3K9ac) |
| *Tet2* | F: TCACCATGCCTGACCTAAAGC | ChIP-qPCR |
|  | R: CCAATTGTTGAAAACGCTGTCT | (H3K27ac) |
| *Cfl2* | F: CCCGTAATGAGATCTGAC | ChIP-qPCR |
|  | R: TATACAAACCATCACAACGC | (H3K27ac) |


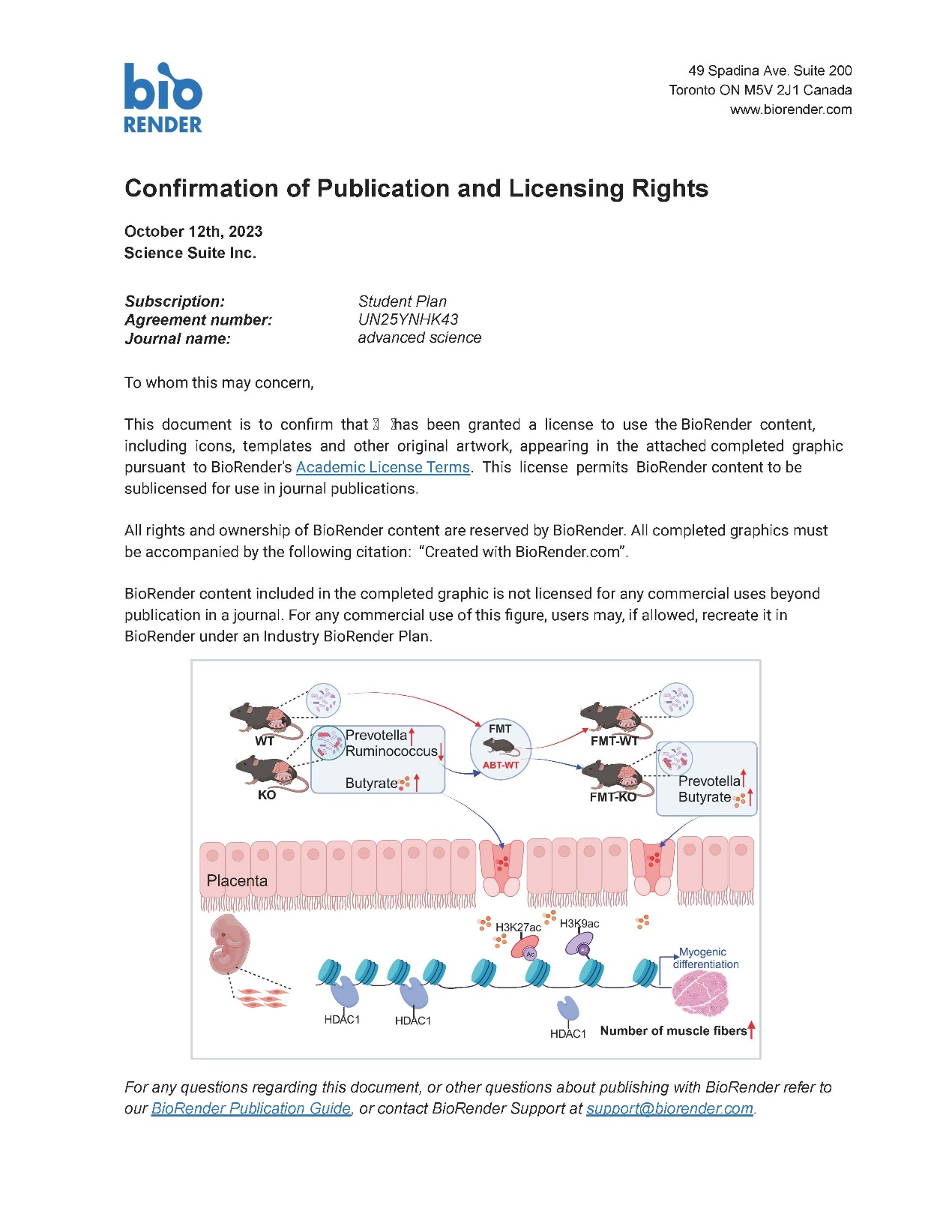

Supplement: Supplementary file 1 — Supporting Information [file ADVS-12-2410953-s001.docx]
